# Temperature-Dependent Lipid Accumulation in the Polar Marine Microalga *Chlamydomonas malina* RCC2488

**DOI:** 10.3389/fpls.2020.619064

**Published:** 2020-12-23

**Authors:** Daniela Morales-Sánchez, Peter S. C. Schulze, Viswanath Kiron, Rene H. Wijffels

**Affiliations:** ^1^The Norwegian College of Fishery Science, Faculty of Biosciences, Fisheries and Economics, UiT – The Arctic University of Norway, Tromsø, Norway; ^2^Faculty of Biosciences and Aquaculture, Nord University, Bodø, Norway; ^3^Green Colab – Associação Oceano Verde, University of Algarve, Faro, Portugal; ^4^Bioprocess Engineering, AlgaePARC, Wageningen University, Wageningen, Netherlands

**Keywords:** psychrophilic microalgae, oleaginous, PUFA, TAG, temperature

## Abstract

The exploration of cold-adapted microalgae offers a wide range of biotechnological applications that can be used for human, animal, and environmental benefits in colder climates. Previously, when the polar marine microalga *Chlamydomonas malina* RCC2488 was cultivated under both nitrogen replete and depleted conditions at 8°C, it accumulated lipids and carbohydrates (up to 32 and 49%, respectively), while protein synthesis decreased (up to 15%). We hypothesized that the cultivation temperature had a more significant impact on lipid accumulation than the nitrogen availability in *C. malina*. Lipid accumulation was tested at three different temperatures, 4, 8, and 15°C, under nitrogen replete and depleted conditions. At 4°C under the nitrogen replete condition *C. malina* had the maximal biomass productivity (701.6 mg L^–1^ day^–1^). At this condition, protein content was higher than lipids and carbohydrates. The lipid fraction was mainly composed of polyunsaturated fatty acids (PUFA) in the polar lipid portion, achieving the highest PUFA productivity (122.5 mg L^–1^ day^–1^). At this temperature, under nitrogen deficiency, the accumulation of carbohydrates and neutral lipids was stimulated. At 8 and 15°C, under both nitrogen replete and depleted conditions, the lipid and carbohydrate content were higher than at 4°C, and the nitrogen stress condition did not affect the algal biochemical composition. These results suggest that *C. malina* is a polar marine microalga with a favorable growth temperature at 4°C and is stressed at temperatures ≥8°C, which directs the metabolism to the synthesis of lipids and carbohydrates. Nevertheless, *C. malina* RCC2488 is a microalga suitable for PUFA production at low temperatures with biomass productivities comparable with mesophilic strains.

## Introduction

In recent years, it has been demonstrated that several microalgal species are efficient for lipid production (Guschina and Harwood, 2006; Da Silva et al., 2009; Harwood and Guschina, 2009; Li-Beisson et al., 2015). Products from microalgal lipids include polyunsaturated fatty acids (PUFA), pigments, antioxidants, and neutral lipids, among others (Grima et al., 1995; Spolaore et al., 2006; Chisti, 2008; Gong et al., 2011; Milledge, 2011; Andruleviciute et al., 2014; Tiwari and Kiran, 2018). Consequently, the processes that have been developed to obtain these compounds usually involve the cultivation of high-lipid content microalgae, or the use of strategies to increase intracellular lipid content (Rodolfi et al., 2009; Pal et al., 2011; He et al., 2015; Hulatt et al., 2017b). Some of these strategies include the control of nutrient availability (carbon, nitrogen, phosphorous and silicate) (Xin et al., 2010; Msanne et al., 2012; Jiang et al., 2014), the manipulation of environmental conditions (pH, salinity, light intensity, and temperature) (Renaud et al., 2002; Converti et al., 2009; Pal et al., 2011; Liu et al., 2012; Draaisma et al., 2013), and genetic and metabolic engineering approaches (Courchesne et al., 2009; Radakovits et al., 2010; Liu and Benning, 2013; Li-Beisson et al., 2015). *Nannochloropsis gaditana* is a mesophilic microalga which is often used for lipid production and upon nitrogen stress conditions it is increasing the lipid content (Hulatt et al., 2017b). High light intensities (600 μmol photons m^–2^ s^–1^) stimulated the lipid accumulation in *Chlorella sorokoniana, C. viscosa*, *C. emersoni*, and *C. vulgaris* (Takeshita et al., 2014). The cold-adapted microalga *Koliella antarctica* showed PUFA increase under phosphorous depletion (Suzuki et al., 2018). Low temperature cultivation stimulated PUFA synthesis in *Scenedesmus* sp. (Xin et al., 2011).

In our previous study, the polar microalga *Chlamydomonas malina* RCC2488 (*C. malina* hereafter) was tested for lipid accumulation using a nitrogen stress strategy. The growth temperature suggested was 8°C (Schulze et al., 2019) which yielded high biomass and lipid productivities. Interestingly, we observed that under both, nitrogen replete and deplete conditions, *C. malina* cells accumulated lipids and carbohydrates, while protein synthesis decreased (Morales-Sánchez et al., 2020). It is rather unique for microalgae to accumulate lipids and carbohydrates under nutrient replete conditions. At these conditions, most microalgae cells proliferate and duplicate, biosynthesizing mainly proteins (Guccione et al., 2014; Barka and Blecker, 2016; Bleakley and Hayes, 2017; Hulatt et al., 2017b). We hypothesize that *C. malina* was in a temperature stress condition at 8°C, since this strain is a polar microalga with an optimum temperature below 8°C (Balzano et al., 2012), which lead to lipids and carbohydrate accumulation. To address this hypothesis, *C. malina* was cultivated at three different temperatures (4, 8, and 15°C) under nitrogen replete and deplete conditions. Growth kinetics, biomass productivities and macromolecular composition are reported here. Non-polar lipid accumulation in *C. malina* was monitored in a fluorescence microscope by staining cells with the fluorophore Nile red.

## Materials and Methods

### Strain and Culture Conditions

The *C. malina* RCC2488 (strain *Chlamydomonas* sp. MALINA FT89.6 PG5, Roscoff Culture Collection, henceforth referred to as *C. malina*) is a marine microalga isolated from the Beaufort Sea, within the Arctic Ocean (Balzano et al., 2012). The maintenance of the algal stock was made in agar plates containing f/2 medium (Guillard and Ryther, 1962). For all experiments, inocula were prepared in 250 mL shake-flaks containing 100 mL of f/2 medium as detailed earlier (Morales-Sánchez et al., 2020). The preparation of f/2 medium included the use of seawater from the North Atlantic shoreline of Bodø (Norway) with a salinity of approximately 35, which was adjusted to a salinity concentration of 17.5 using distilled water for optimal growth, according to a previous study (Morales-Sánchez et al., 2020). All experiments were conducted in bubble columns photobioreactors (Hulatt et al., 2017a; Morales-Sánchez et al., 2020). The reactors were inoculated with 0.2 g of dry cell weight (DCW) per L (g_*DCW*_ L^–1^) and operated at three different temperatures (4, 8, and 15°C). Cells were cultured until mid-exponential phase (5 days) in f/2 medium and 120 μmol photons m^–2^ s^–1^ of light intensity. After this period, cells were collected by centrifugation, washed twice and resuspended in f/2 medium with (a) nitrogen replete or (b) nitrogen deplete (here abbreviated to as +N and −N, respectively) conditions for cultivation in the bubble columns. These experiments were maintained at 120 μmol photons m^–2^ s^–1^ of light intensity during 3 days. At the end of the experimental period, cells were harvested by centrifugation at 2,000 *g* for 5 min, washed with 0.5 M ammonium formate, centrifuged again as before and the pellets were stored at −70°C for further analyses. All experiments were carried out in triplicate.

### Growth Measurements

The cellular growth was determined based on the dry cell weight (DCW) measurement. A correlation was made between the optical cell density and the dry weight using the equation:

(1) $$W=\left(0.884\times A_{750}\right)+0.0117$$

Where *W* is the DCW (g_*DCW*_ L^–1^) and *A*_750_ is the absorbance measured at 750 nm. Culture samples (0.5–1 mL) were collected daily to measure the absorbance at 750 nm in a 1 cm micro-cuvette using a spectrophotometer (Hach-Lange DR3900, Hach, International). The algal DCW was evaluated gravimetrically by filtrating 5–10 mL of culture broth through a pre-dried and pre-weighed 0.45 μm pore size nitrocellulose membrane filter (Merck Milipore, MA, United States). The membrane containing the biomass was washed three times with 0.5 M ammonium formate prior drying in an oven at 60°C for 24 h or until constant weight.

### Lipid, Protein, and Carbohydrate Analyses

Organic solvents, such as chloroform and methanol, were used to extract lipids from *C. malina*. Fatty acid methyl esters (FAMEs) from both, neutral and polar lipids were identified by gas chromatography (GC) following the methodology fully described previously (Breuer et al., 2013; Morales-Sánchez et al., 2020). Solid-phase extraction was used to separate neutral and polar lipids (Hulatt et al., 2017a). Briefly, total lipid extracts were dissolved in 0.5 mL hexane:diethyl ether (7:1 v/v) and loaded onto 6 mL (1 g) silica cartridges (Supelco^®^). Neutral lipids were first eluted with 10 mL of hexane:diethyl ether (7:1 v/v), then 10 mL of methanol:acetone:hexane (2:2:1 v/v/v) was added to elute the polar lipids. A stream of nitrogen gas was used to dry each lipid fractions. The fatty acyl chains contained in both lipid fractions were derivatized to FAMEs and identified by GC using the methodology previously mentioned (Breuer et al., 2013; Hulatt et al., 2017a; Morales-Sánchez et al., 2020).

Neutral lipids content in *C. malina* was detected by fluorescence microscopy as previously described (Msanne et al., 2012; Morales-Sánchez et al., 2017). Briefly, *C. malina* cells were stained with the lipophilic fluorophore Nile Red, incubated at 20°C for 15 min and visualized in a fluorescence microscope (Biorad Zoe^TM^, CA, United States).

Carbohydrate analysis was performed using the phenol-sulfuric acid method (Thompson, 1950) after hydrolysis of the biomass using 1 N HCl to produce reducing sugars.

Protein content was analyzed using the Lowry method (Lowry et al., 1951) after alkaline hydrolysis of the sample with 1 N NaOH.

### Calculations

The cellular growth kinetics and productivities were calculated accordingly with a 4-parameter logistic function (Hulatt et al., 2017b), following the next equations:

(2) $$C_{x}=\emptyset 1+\frac{\emptyset 2-\emptyset 1}{1+\text{exp}\left(\frac{\emptyset 3-t}{\emptyset 4}\right)}$$

Where *C*_*x*_ is the DCW (g_*DCW*_ L^–1^) at time t (days), φ1 is the lowest asymptote (minimum *C*_*x*_), φ2 is the upper asymptote (maximum C_*x*_), φ3 is t at 0.5φ2 (the inflection point) and φ4 is the scale parameter (Hulatt et al., 2017b). Consequently, the productivity was calculated between two time points, accordingly with the next equation (Eq. 3):

(3) $$P_{i}=\frac{C_{x,i}-C_{x,i-1}}{t_{i}-t_{i-1}}$$

Where *P*_*i*_ is the productivity (g_*DCW*_ L^–1^ day^–1^), *C*_*x*_,_*i*_ and *C*_*x*_,_*i*__–__1_ are the concentrations of the biomass (g_*DCW*_ L^–1^) at two time points and *t*_*i*_ and *t*_*i*__–__1_ are the time of cultivation (days). The specific cellular growth rate *k* (d^–1^) was therefore derived (Eq. 4):

(4) $$k_{i}=\frac{P_{i}}{C_{X,1}}$$

The duplication time (t_*D*_), which is the time to double the number of cells, was then calculated from the specific growth rate (Eq. 4):

(5) $$t_{D}=\frac{ln2}{k_{i}}$$

### Statistical Analysis

Shapiro-Wilk test was used to validate the normal distribution of the data, and Brown-Forsythe test was applied to confirm the homogeneity of the variance between treatments. One-way analysis of variance (ANOVA) and *post hoc* Tukey’s multiple comparison test was applied to each set of experiments in order to determine statistical differences among treatments. *P* values smaller than 0.05 were considered statistically significant. Details of each test are described in Supplementary Material Statistical Procedures 1.

## Results

### Effect of Temperature and Nitrogen Availability on the Growth and Productivity of *Chlamydomonas malina*

**FIGURE 1:**
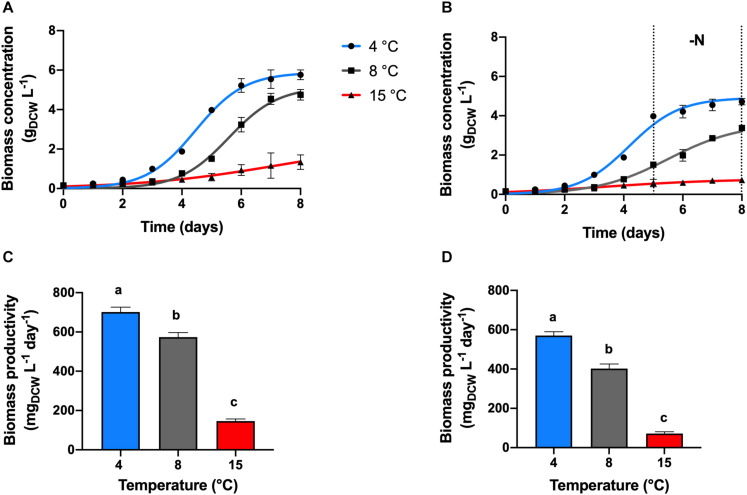
Growth kinetics and productivity. Effect of temperature on the growth kinetics and productivities of *C. malina* under +N **(A,C)** and –N **(B,D)** conditions, after 8 days of batch cultivation in bubble-column photobioreactors. Data are presented as means ± SD of three independent experiments. Dotted black lines denote the nitrogen deprivation period. Different lowercase letters indicate a significant difference among means of groups (one-way ANOVA with *post hoc* Tukey HSD test, *p* < 0.05).

Maximal biomass concentration of 5.61 g_*DCW*_ L^–1^ and productivity of 701.6 mg_*DCW*_ L^–1^ day^–1^ were found at a temperature cultivation of 4°C, in the nitrogen replete condition (+N, Figures 1A,C, *p* < 0.05). *Chlamydomonas malina* cells grew at a duplication time (t_*D*_) of 11 h at 4°C, which was 6 and 71 h faster, compared with 8 and 15°C, respectively (*p* < 0.05). At 15°C, cells showed a linear growth, the biomass productivity decreased significantly about 5 and 4-times compared with 4 and 8°C, respectively (*p* < 0.05).

Under nitrogen deprivation (−N), cell growth was arrested in all treatments compared with +N conditions. After a period of nutrient sufficiency during the first 5 days of cultivation, cells entered to a nitrogen starvation period in which cells grew slower in all temperature tested (Figure 1B). During the 8 days of cultivation at 4°C in the −N treatment, the total cell concentration reached 4.6 g_*DCW*_ L^–1^, with an overall productivity of 570 mg_*DCW*_ L^–1^ day^–1^ (Figure 1D). These values represent about 20% decreased compared with the values obtained at +N conditions (*p* < 0.05). At these conditions, the analysis of the starvation period (3 days) showed that the cell concentration increased only 18% (DCW) in that time, which represents a biomass productivity of 24.5 mg_*DCW*_ L^–1^ day^–1^. At 8°C, cells presented a linear biomass accumulation during the 8 days of cultivation in −N, the biomass concentration and productivity were reduced in about 30% compared to +N conditions (Figures 1B,D; *p* < 0.05). However, the productivity during the starvation period was 2.5-times higher (62.4 mg_*DCW*_ L^–1^, *p* < 0.05) compared with the productivity at 4°C. At 15°C under −N, biomass concentration and productivity were the lowest of all temperature treatments (Figures 1B,D; *p* < 0.05). At these conditions (15°C, −N), the overall biomass concentration and productivity during 8 days were 2-times lower than cells maintained at same temperature and +N conditions (*p* < 0.05). During the starvation period, the biomass concentration increased 26% (DCW), and the productivity was 5.15 g_*DCW*_ L^–1^, which was the lowest productivity obtained in this work.

### Effect of Temperature and Nitrogen Availability on the Biochemical Composition of *Chlamydomonas malina*

#### Protein

**FIGURE 2:**
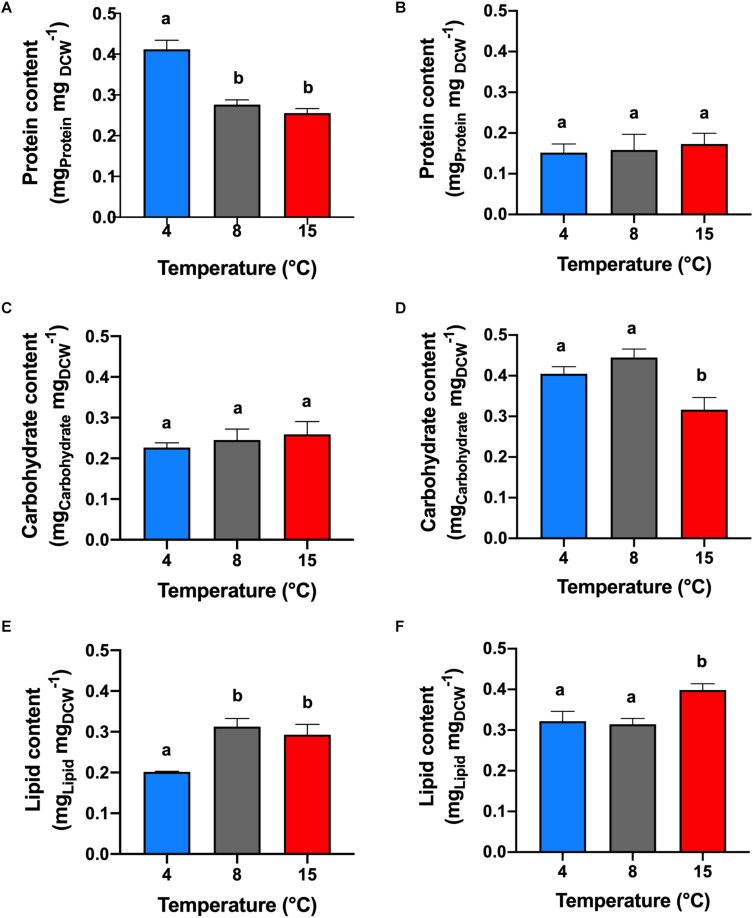
Protein, carbohydrate and lipid contents. Effect of temperature on the protein **(A,B)**, carbohydrate **(C,D)**, and lipid **(E,F)** contents of *C. malina* under +N **(A,C,E)** and –N **(B,D,F)** conditions in bubble-column photobioreactors. Samples in the +N condition were taken in the middle exponential growth, and in the –N condition after 3 days of starvation. Data are presented as means ± SD of three independent experiments. Different lowercase letters indicate a significant difference among means of groups (one-way ANOVA with *post hoc* Tukey HSD test, *p* < 0.05).

Under +N conditions (Figure 2A), the analysis of protein content was made in the middle of the exponential phase (5th cultivation day). The highest protein content of 41% (mg_*protein*_ mg_*DCW*_^–1^) was obtained in cells cultivated at 4°C (*p* < 0.05). The protein content in the treatments at 8 and 15°C were not significantly different (*p* > 0.05) between each other with an average of 26.5%.

Under −N conditions (Figure 2B), the protein determination was performed after 3 days of nitrogen starvation (8th cultivation day). The protein content among the temperature treatments were not statistically different (*p* > 0.05), with an average of 16% of protein. However, at this condition, the protein content decreased in all treatments, compared with cells cultivated under +N conditions (*p* < 0.05). The reduction in protein content was significant at 4°C (25% reduction), and less at 8°C (12%), and at 15°C (8%).

#### Carbohydrates

Cells cultivated at +N conditions (Figure 2C), during exponential growth, had similar carbohydrate content (*p* > 0.05), obtaining in average 24.4% (mg_*Carbohydrate*_ mg_*DCW*_^–1^). However, cells maintained at −N (3 days after mid-exponential growth; Figure 2D) presented higher carbohydrate content compared with cells cultivated in +N conditions (*p* < 0.05). Highest carbohydrate content was obtained in cells cultivated at 4 and 8°C, obtaining on average 42.3% with no significant differences among them (*p* > 0.05).

#### Lipids

**FIGURE 3:**
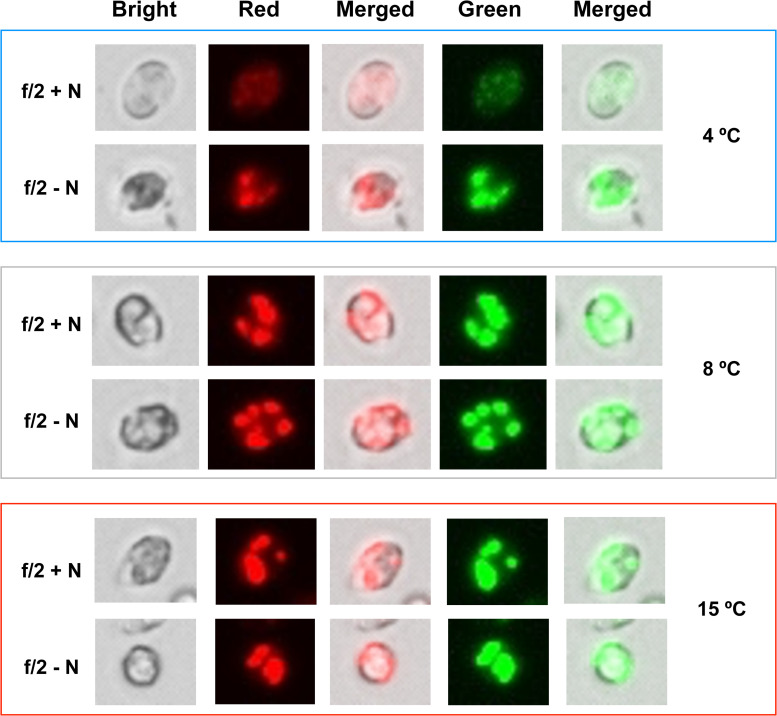
Neutral lipid stained with Nile Red. Neutral lipid accumulation in *C. malina* cells cultivated at 4, 8, and 15°C subjected to +N and –N conditions. Cells at different temperatures were cultured for 2 days in f/2 medium at +N and –N conditions, harvested and stained with Nile Red. Lipid bodies were detected in a fluorescent microscopy in three different filters (bright, red and green). The images shown are representative of typical cells in the samples. Scale bar can be seen in Supplementary Figure 1.

Cells maintained at 4°C under +N conditions showed lipid content of 20% (mg_*LIPID*_ mg_*DCW*_^–1^), which was the lowest value reported in this study. Under these conditions, formation of lipid bodies was not detected by Nile Red fluorescence (Figure 3). However, lipid content in cells cultivated at 8 and 15°C in +N conditions presented an unusual behavior (Figure 2E). At these temperatures, the lipid content was extraordinarily high (30%), with no significant differences between these temperature treatments (*p* > 0.05). In addition, Nile Red fluorescence indicated high amounts of neutral lipids in cells exposed to 8 and 15°C (Figure 3). In all temperature treatments at +N condition, cells for lipid (as well as for protein and carbohydrate) analysis were taken in the mid-exponential growth phase, ensuring nutrient-sufficient conditions. The lipid content in cells cultivated under −N at 4 and 8°C showed no significant difference (*p* > 0.05), obtaining up to 31.8% in average (Figure 2F).

Under −N condition, maximum lipid content was found at 15°C, reaching 40%. At 4°C, the lipid content increased 12% in cells cultivated under −N, compared with cells maintained at +N conditions. At 8°C, the lipid content was not significantly different (*p* > 0.05) in cells cultivated at +N and −N conditions. The lipid content increased 10% in cells cultivated at 15°C under −N, related to cells grown in +N conditions. Nile Red fluorescence revealed high lipid body formation in cells under −N in all temperatures tested (Figure 3).

#### Fatty Acid Content and Profiles

**FIGURE 4:**
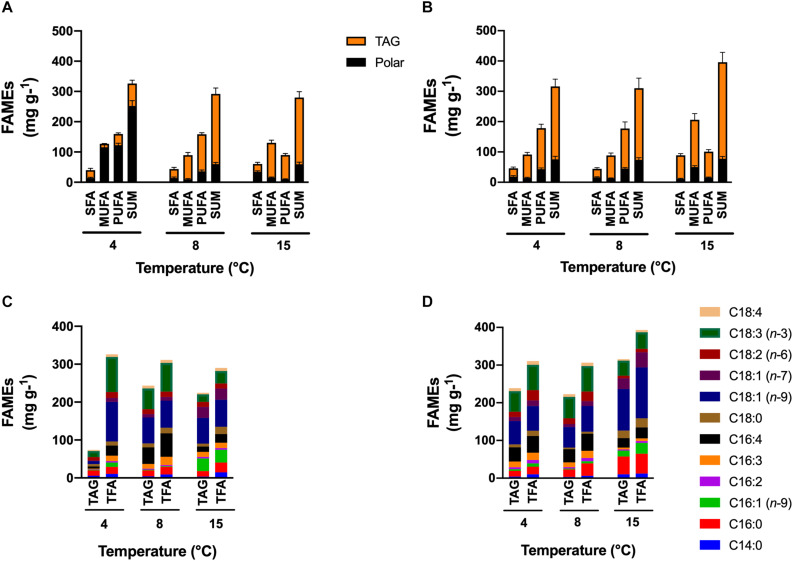
FAMEs content and profile. Effect of temperature on the FAMEs content and profile of total fatty acids (TFA) and fatty acids in triacylglycerols (TAG) contained in *C. malina* under the +N **(A,C)** and the –N **(B,D)** conditions in tubular photobioreactors. Samples in the +N condition were taken in the middle exponential growth, and in the –N condition after 3 days of starvation. SFA, saturated fatty acids; MUFA, monounsaturated fatty acids; PUFA, polyunsaturated fatty acids; TAG, triacylglycerols. Data are presented as means ± SD of three independent experiments. Statistical comparison was performed individually for each component and each class of fatty acid (polar, TAGs, SFA, MUFA, PUFA, and sum) among the treatments.

Under +N conditions, the major lipid content was found in the polar lipid fraction for cells maintained at 4°C, and in the TAG fraction for cells at 8 and 15°C. The saturated fatty acid content (SFA) in the polar fraction was higher in cells at 15°C (34.4 mg g^–1^; *p* < 0.05), but in the TAG fraction, the SFA content had no significant differences among all temperature treatments (Figure 4A). Cells at 4°C had increased monounsaturated fatty acid (MUFA) in the polar fraction (115.5 mg g^–1^) but decreased in the TAG fraction (11.4 mg g^–1^). Contrary to cells at 4°C, cells at higher temperatures had decreased MUFA content in the polar fraction and increased in the TAG fraction. Highest PUFA content was found in cells cultivated at 4°C in the polar fraction (122.5 mg g^–1^, *p* < 0.05) and cells cultivated at 8 and 15°C in the TAG fraction (122 and 80 mg g^–1^, respectively).

Cells maintained under −N in all temperatures accumulated preferentially TAG as the major lipid content (Figure 4B). In the polar fraction, the SFA content was similar in all treatments. The MUFA content was significantly higher in cells cultivated at 15°C (4-times; *p* < 0.05) compared to cells maintained at lower temperatures. The opposite effect was observed for the PUFA content, cells at lower temperature had significantly higher content (3-times; *p* < 0.05) compared with cells at 15°C. In the TAG fraction, the SFA content was higher in cells at 15°C (3-times; *p* < 0.05). The same effect observed in the polar fraction was also noted in the TAG fraction for the MUFA and PUFA content. The MUFA content was higher in cells at 15°C (2-times; *p* < 0.05), but the PUFA content was higher in cells at 4 and 8°C (1.5-times; *p* < 0.05). The highest PUFA productivity under −N conditions was found at 4°C (79.7 mg L^–1^ day^–1^; *p* < 0.05).

Under +N conditions at 4°C, cells mainly synthesized PUFA, such as hexadecatetraenoic acid (C16:4*n*-3) and α-linolenic acid (C18:3*n*-3), and the MUFA oleic acid (C18:1*n*-9) in the polar fraction (Figure 4C). Cells maintained at 8°C synthesized the same fatty acid classes than at 4°C, but they were found mainly in the TAG fraction. At 15°C, the major fatty acid types were MUFA in both fractions, like palmitoleic acid (C16:1*n*-7), C18:1*n*-9 and vaccenic acid (C18:1*n*-7). Saturated fatty acids such as palmitic acid (C16:0) and stearic acid (C18:0) were found in cells cultivated at 15°C in higher proportion (*p* < 0.05) than in cells at lower temperatures. Under −N conditions (Figure 4D), TAG fraction contained the majority of lipids in all treatments. In general, SFA such as C16:0 and C18:0, and MUFA like C18:1*n*-9 and C18:1*n-7* were found abundantly in cells cultivated at 15°C. At lower temperatures (4 and 8°C), C18:1*n*-9 and C18:3*n*-3 were mainly found.

## Discussion

Most microalgal species are able to grow and accomplish photosynthesis over a wide range of temperatures, with optimal conditions between 20 and 25°C (Li, 1980; Ras et al., 2013). These species are called mesophilics and their metabolism responds to the exponential (Q_10_) or the Arrhenius equation, in which it is stated that below optimal growth temperatures, an increase in the temperature has a positive effect on photosynthesis and growth (Ahlgren, 1987; Ras et al., 2013).

On the other hand, there are some microalgae found in the polar and cold regions known as psychrophiles, which have optimum growth temperatures at or below 15°C (Morgan-Kiss et al., 2006). These microalgae have experienced a strong and persistent selection and have evolved to develop a wide range of physiological adaptations that allow them to thrive at temperatures close to or below freezing (Morgan-Kiss et al., 2006; Hulatt et al., 2017a). The strain *C. malina* RCC2488 is a psychrophilic alga isolated from the Beufourt Sea within the Arctic Ocean (Balzano et al., 2012). This microalga has its highest growth rate and biomass productivity at low temperatures (4°C). Microalgae should invariably reduce its metabolic rate at cold conditions (Hulatt et al., 2017a), but as demonstrated by *C. malina* in this study and other cold-adapted algal strains (Lyon and Mock, 2014; Cao et al., 2016; Hulatt et al., 2017a; Suzuki et al., 2018), this is not applicable to microalgae found living in polar or cold environments. At temperatures exceeding the optimum, a decrease in the microalgal growth rate can be observed as a response to heat stress, which can affect the metabolism thereby inhibiting growth (Ras et al., 2013).

Microalgae adapted to cold environments might be stressed at temperatures above the optimum. This stress may be reflected on its macromolecular composition, as in the case of *C. malina* which synthesized high lipid and carbohydrate content at 8 and 15°C, even under nutrient replete conditions (+N). This phenomenon is unusual and remarkable since normally microalgal cells respond to nutrient sufficiency conditions by synthesizing the cellular building blocks proteins (Morales-Sánchez et al., 2013), as observed in *C. malina* cultivated at 4°C. However, at 8 and 15°C, in both +N and −N conditions, the protein content was lower compared to the high lipid and carbohydrate content, suggesting a stress condition caused by temperature. Therefore, nitrogen stress did not cause an effect on the biochemical composition of the cells at 8 and 15°C, but it caused the usual phenomenon of lipid accumulation at 4°C under nitrogen deprivation (−N), as observed in many mesophilic algae in nitrogen-deprived cultivations (Pruvost et al., 2009; Lv et al., 2010; Work et al., 2010; Msanne et al., 2012; Li-Beisson et al., 2013; Liu and Benning, 2013; Çakmak et al., 2014). Also, at higher temperatures (8 and 15°C), cells mainly synthesized neutral lipids (TAG), probably as a consequence of stress. However, cells cultivated at 4°C and +N conditions mainly synthesized PUFA in the polar fraction (cellular membrane). The reason is that PUFA are essential to keep the fluidity of the membrane at low temperatures (D’Amico et al., 2006; Morgan-Kiss et al., 2008). Especially, polyunsaturated and short-chain length fatty acids, such as C16:4*n-*3 which was found in high concentrations in *C. malina*. This C16:4*n-*3 acyl group is nearly exclusively present in monogalactosyldiacylglycerol (MGDG) –the most abundant membrane lipid in Chlamydomonas chloroplast– and is known to be a significant contributor in the transition from liquid-crystalline to gel phase (Dolhi et al., 2013; Li-Beisson et al., 2013; Liu and Benning, 2013). It is hypothesized that the availability of C16:4*n-*3 affects the total amount of the prevalent MGDG molecular species that contains C18:3*n-*3 in the *sn-1* and C16:4*n-*3 in the *sn-2* position of the glycerol backbone (Liu and Benning, 2013). It is still unknown why C16:4*n-*3 is primarily present in MGDG and how its abundance is regulated in this membrane galactolipid. Therefore, it has been suggested that loss-of-function studies with mutants entirely lacking (or decreased amounts) 16:4*n-*3 could be interesting to possibly answer what are the specific roles of this molecular species of MGDG in the photosynthetic membrane (Liu and Benning, 2013). Similar to our findings, the same classes of fatty acids were found in the polar microalga and closest *C. malina* relative, UWO 241 (Morgan-Kiss et al., 2002). Cells of *C. malina* cultivated at 4°C responded in a similar way to other algal species when they were cultivated at +N conditions by stimulating the synthesis of proteins, and to −N conditions by accumulating lipids and carbohydrates, and reducing nitrogen-rich compounds like proteins (Siaut et al., 2011; Msanne et al., 2012; Morales-Sánchez et al., 2013; Schmollinger et al., 2014; Zhu et al., 2016). Also, under −N, the lipid fraction was mainly composed of TAG.

Our results indicate that *C. malina* can be highly productive at low temperatures, making it a potential candidate for biomass, carbohydrate and polyunsaturated lipids production in cold environments. Table 1 present a comparison of biomass productivity of several mesophilic and polar/cold-adapted microalgae, including *C. malina* at best growth conditions. As observed, *C. malina* productivity is comparable with mesophilic microalgae and high productive polar/cold-adapted microalgae.

**TABLE 1:** Analysis of biomass productivities.

Microalgal strain	Productivity (mg L^–1^ day^–1^)	Temperature (°C)	References
*Chlamydomonas malina*	701	8	This study
*Chlamydomonas pulsatilla*	580	6	Hulatt et al., 2017a
*Chloromonas platystigma*	250	6	Hulatt et al., 2017a
*Chlamydomonas klinobasis*	215	6	Hulatt et al., 2017a
*Raphidonema sempervirens*	133	6	Hulatt et al., 2017a
*Koliella antarctica*	480	15	Suzuki et al., 2018
*Koliella antarctica*	2,370	15	Suzuki et al., 2018
*Nannochloropsis gaditana*	(best condition)	25	
	510		Hulatt et al., 2017b
*Chlorella* sp. F&M-M49	640	25	Chen et al., 2017
*Chlorella* sp. CCAP 211-11b	590	25	Chen et al., 2017
*Chlorella* sp. IAM C-212	710	25	Chen et al., 2017
*Chlorella* sp. PROD1	730	25	Chen et al., 2017

*Comparison of biomass productivities of C. malina cultivated at 4°C, 17.5 salinity, nitrogen replete conditions and 120 μmol m^–2^ s^–1^ of light intensity with other polar and cold-adapted microalgae strains cultivated at similar conditions and mesophilic microalgae cultivated at optimum conditions (Chen et al., 2017; Hulatt et al., 2017a, b; Suzuki et al., 2018).*

## Conclusion

Polar *C. malina* is a marine microalga that can be stressed at temperatures ≥8°C. As a consequence, high accumulation of carbohydrates and lipids can be stimulated. Particularly, the lipid fraction was predominantly composed of TAG with a high PUFA productivity of 122 mg L^–1^ day^–1^. At these temperatures, nitrogen stress did not have an effect on the algal biochemical composition. At 4°C, under +N conditions, *C. malina* synthesized mainly proteins and the lipid portion was primarily found in the polar fraction with high PUFA content (122.5 mg L^–1^ day^–1^). At this temperature, −N stimulated the synthesis of lipids but arresting the cell growth and protein synthesis. Nevertheless, the PUFA productivity was high as well, reaching up to 79.7 mg L^–1^ day^–1^. Under −N, carbohydrate content increased between 32 and 44% in all temperature tested. Neutral lipids increased at 15°C, reaching the highest content of this study (44% DCW). The polar microalga *C*. *malina* has high potential for carbohydrates, lipids and PUFA production in cold climates, with biomass productivities comparable with mesophilic microalgae.

## Data Availability Statement

The original contributions presented in the study are included in the article/Supplementary Material, further inquiries can be directed to the corresponding author/s.

## Author Contributions

Within the work package in the A2F project, RW designed the research that resulted in this manuscript. DM-S designated the study, collected the data, and conducted the bioreactor experiments. PS performed the lipid and fatty acid analysis. DM-S, PS, VK, and RW contributed to manuscript drafting, discussion, and critical revision of the article for important intellectual content. All authors contributed to the article and approved the submitted version.

## Conflict of Interest

The authors declare that the research was conducted in the absence of any commercial or financial relationships that could be construed as a potential conflict of interest.

## References

[B1] Ahlgren G. (1987). Temperature Functions in Biology and Their Application to Algal Growth Constants. *Oikos* 49:177 10.2307/3566025

[B2] Andruleviciute V. Makareviciene V. Skorupskaite V. Gumbyte M. (2014). Biomass and oil content of Chlorella sp., Haematococcus sp., Nannochloris sp. and Scenedesmus sp. under mixotrophic growth conditions in the presence of technical glycerol. *J. Appl. Phycol.* 26 83–90. 10.1007/s10811-013-0048-x

[B3] Balzano S. Gourvil P. Siano R. Chanoine M. Marie D. Lessard S. (2012). Diversity of cultured photosynthetic flagellates in the northeast Pacific and Arctic Oceans in summer. *Biogeosciences* 9 4553–4571. 10.5194/bg-9-4553-2012

[B4] Barka A. Blecker C. (2016). Microalgae as a potential source of single-cell proteins. *Biotechnol. Agron. Soc. Environ.* 20 427–436.

[B5] Bleakley S. Hayes M. (2017). Algal Proteins: Extraction, Application, and Challenges Concerning Production. *Foods* 6:33. 10.3390/foods6050033 28445408 PMC5447909

[B6] Breuer G. Evers W. A. C. de Vree J. H. Kleinegris D. M. M. Martens D. E. Wijffels R. H. (2013). Analysis of fatty acid content and composition in microalgae. *J. Vis. Exp.* 2013:50628. 10.3791/50628 24121679 PMC3938209

[B7] Çakmak Z. E. Ölmez T. T. Çakmak T. Menemen Y. Tekinay T. (2014). Induction of triacylglycerol production in *Chlamydomonas reinhardtii*: Comparative analysis of different element regimes. *Bioresour. Technol.* 155 379–387. 10.1016/j.biortech.2013.12.093 24472680

[B8] Cao K. He M. Yang W. Chen B. Luo W. Zou S. (2016). The eurythermal adaptivity and temperature tolerance of a newly isolated psychrotolerant Arctic *Chlorella* sp. *J. Appl. Phycol.* 28 877–888. 10.1007/s10811-015-0627-0

[B9] Chen B. Wan C. Mehmood M. A. Chang J.-S. Bai F. Zhao X. (2017). Manipulating environmental stresses and stress tolerance of microalgae for enhanced production of lipids and value-added products–A review. *Bioresour. Technol.* 244 1198–1206. 10.1016/j.biortech.2017.05.170 28601395

[B10] Chisti Y. (2008). Biodiesel from microalgae beats bioethanol. *Trends Biotechnol.* 26 126–131. 10.1016/j.tibtech.2007.12.002 18221809

[B11] Converti A. Casazza A. A. Ortiz E. Y. Perego P. Del Borghi M. (2009). Effect of temperature and nitrogen concentration on the growth and lipid content of *Nannochloropsis oculata* and *Chlorella vulgaris* for biodiesel production. *Chem. Eng. Process. Process Intensif.* 48 1146–1151. 10.1016/j.cep.2009.03.006

[B12] Courchesne N. M. D. Parisien A. Wang B. Lan C. Q. (2009). Enhancement of lipid production using biochemical, genetic and transcription factor engineering approaches. *J. Biotechnol.* 141 31–41. 10.1016/j.jbiotec.2009.02.018 19428728

[B13] D’Amico S. Collins T. Marx J.-C. Feller G. Gerday C. (2006). Psychrophilic microorganisms: challenges for life. *EMBO Rep.* 7 385–389. 10.1038/sj.embor.7400662 16585939 PMC1456908

[B14] Da Silva T. L. Reis A. Medeiros R. Oliveira A. C. Gouveia L. (2009). Oil production towards biofuel from autotrophic microalgae semicontinuous cultivations monitorized by flow cytometry. *Appl. Biochem. Biotechnol.* 159 568–578. 10.1007/s12010-008-8443-5 19067244

[B15] Dolhi J. M. Maxwell D. P. Morgan-Kiss R. M. (2013). The Antarctic *Chlamydomonas raudensis*: An emerging model for cold adaptation of photosynthesis. *Extremophiles* 17 711–722. 10.1007/s00792-013-0571-3 23903324

[B16] Draaisma R. B. Wijffels R. H. Breuer G. Lamers P. P. Martens D. E. (2013). Effect of light intensity, pH, and temperature on triacylglycerol (TAG) accumulation induced by nitrogen starvation in *Scenedesmus obliquus*. *Bioresour. Technol.* 143 1–9. 10.1016/j.biortech.2013.05.105 23774290

[B17] Gong Y. Hu H. Gao Y. Xu X. Gao H. (2011). Microalgae as platforms for production of recombinant proteins and valuable compounds: Progress and prospects. *J. Ind. Microbiol. Biotechnol.* 38 1879–1890. 10.1007/s10295-011-1032-6 21882013

[B18] Grima E. M. Pérez J. A. S. Camacho F. G. Medina A. R. Giménez A. G. López Alonso D. (1995). The production of polyunsaturated fatty acids by microalgae: from strain selection to product purification. *Process Biochem.* 30 711–719. 10.1016/0032-9592(94)00047-6

[B19] Guccione A. Biondi N. Sampietro G. Rodolfi L. Bassi N. Tredici M. R. (2014). *Chlorella* for protein and biofuels: From strain selection to outdoor cultivation in a Green Wall Panel photobioreactor. *Biotechnol. Biofuels* 7:84. 10.1186/1754-6834-7-84 24932216 PMC4057815

[B20] Guillard R. R. Ryther J. H. (1962). Studies of marine planktonic diatoms. I. Cyclotella nana Hustedt, and Detonula confervacea (cleve) Gran. *Can. J. Microbiol.* 8 229–239. 10.1139/m62-029 13902807

[B21] Guschina I. A. Harwood J. L. (2006). Lipids and lipid metabolism in eukaryotic algae. *Prog. Lipid Res.* 45 160–186. 10.1016/j.plipres.2006.01.001 16492482

[B22] Harwood J. L. Guschina I. A. (2009). The versatility of algae and their lipid metabolism. *Biochimie* 91 679–684. 10.1016/j.biochi.2008.11.004 19063932

[B23] He Q. Yang H. Wu L. Hu C. (2015). Effect of light intensity on physiological changes, carbon allocation and neutral lipid accumulation in oleaginous microalgae. *Bioresour. Technol.* 191 219–228. 10.1016/J.BIORTECH.2015.05.021 25997011

[B24] Hulatt C. J. Berecz O. Egeland E. S. Wijffels R. H. Kiron V. (2017a). Polar snow algae as a valuable source of lipids? *Bioresour. Technol.* 235 338–347. 10.1016/j.biortech.2017.03.130 28384586

[B25] Hulatt C. J. Wijffels R. H. Bolla S. Kiron V. (2017b). Production of fatty acids and protein by *Nannochloropsis* in flat-plate photobioreactors. *PLoS One* 12:1–17. 10.1371/journal.pone.0170440 28103296 PMC5245880

[B26] Jiang Y. Laverty K. S. Brown J. Nunez M. Brown L. Chagoya J. (2014). Effects of fluctuating temperature and silicate supply on the growth, biochemical composition and lipid accumulation of *Nitzschia* sp. *Bioresour. Technol.* 154 336–344. 10.1016/j.biortech.2013.12.068 24413451

[B27] Li W. K. W. (1980). “Temperature Adaptation in Phytoplankton: Cellular and Photosynthetic Characteristics,” in *Primary Productivity in the Sea*, ed. Falkowski P. (Boston: Springer), 259–279. 10.1007/978-1-4684-3890-1_15

[B28] Li-Beisson Y. Beisson F. Riekhof W. (2015). Metabolism of acyl-lipids in *Chlamydomonas reinhardtii*. *Plant J.* 82 504–522. 10.1111/tpj.12787 25660108

[B29] Li-Beisson Y. Shorrosh B. Beisson F. Andersson M. X. Arondel V. Bates P. D. (2013). Acyl-Lipid Metabolism. *Arabidopsis Book* 11:e0161. 10.1199/tab.0161 23505340 PMC3563272

[B30] Liu B. Benning C. (2013). Lipid metabolism in microalgae distinguishes itself. *Curr. Opin. Biotechnol.* 24 300–309. 10.1016/j.copbio.2012.08.008 22981869

[B31] Liu J. Yuan C. Hu G. Li F. (2012). Effects of light intensity on the growth and lipid accumulation of microalga *Scenedesmus* sp. 11-1 under nitrogen limitation. *Appl. Biochem. Biotechnol.* 166 2127–2137. 10.1007/s12010-012-9639-2 22415786

[B32] Lowry O. H. Rosebrough N. J. Farr A. L. Randall R. J. (1951). Protein measurement with the Folin phenol reagent. *J. Biol. Chem.* 193 265–275. 14907713

[B33] Lv J. M. Cheng L. H. Xu X. H. Zhang L. Chen H. L. (2010). Enhanced lipid production of Chlorella vulgaris by adjustment of cultivation conditions. *Bioresour. Technol.* 101 6797–6804. 10.1016/j.biortech.2010.03.120 20456951

[B34] Lyon B. Mock T. (2014). Polar Microalgae: New Approaches towards Understanding Adaptations to an Extreme and Changing Environment. *Biology* 3 56–80. 10.3390/biology3010056 24833335 PMC4009763

[B35] Milledge J. J. (2011). Commercial application of microalgae other than as biofuels: A brief review. *Rev. Environ. Sci. Biotechnol.* 10 31–41. 10.1007/s11157-010-9214-7

[B36] Morales-Sánchez D. Kim Y. Terng E. L. Peterson L. Cerutti H. (2017). A multidomain enzyme, with glycerol-3-phosphate dehydrogenase and phosphatase activities, is involved in a chloroplastic pathway for glycerol synthesis in *Chlamydomonas reinhardtii*. *Plant J.* 90 1079–1092. 10.1111/tpj.13530 28273364

[B37] Morales-Sánchez D. Schulze P. S. C. Kiron V. Wijffels R. H. (2020). Production of carbohydrates, lipids and polyunsaturated fatty acids (PUFA) by the polar marine microalga Chlamydomonas malina RCC2488. *Algal Res.* 50:102016 10.1016/j.algal.2020.102016 PMC7785989 33424911

[B38] Morales-Sánchez D. Tinoco-Valencia R. Kyndt J. Martinez A. (2013). Heterotrophic growth of *Neochloris oleoabundans* using glucose as a carbon source. *Biotechnol. Biofuels* 6:100. 10.1186/1754-6834-6-100 23849253 PMC3717095

[B39] Morgan-Kiss R. M. Ivanov A. G. Modla S. Czymmek K. Hüner N. P. A. Priscu J. C. (2008). Identity and physiology of a new psychrophilic eukaryotic green alga, *Chlorella* sp., strain BI, isolated from a transitory pond near Bratina Island, Antarctica. *Extremophiles* 12 701–711. 10.1007/s00792-008-0176-4 18661097

[B40] Morgan-Kiss R. M. Priscu J. C. Pocock T. Gudynaite-Savitch L. Huner N. P. A. (2006). Adaptation and acclimation of photosynthetic microorganisms to permanently cold environments. *Microbiol. Mol. Biol. Rev.* 70 222–252. 10.1128/MMBR.70.1.222-252.2006 16524924 PMC1393254

[B41] Morgan-Kiss R. Ivanov A. G. Williams J. Khan M. Huner N. P. A. (2002). Differential thermal effects on the energy distribution between photosystem II and photosystem I in thylakoid membranes of a psychrophilic and a mesophilic alga. *Biochim. Biophys. Acta* 1561 251–265. 10.1016/S0005-2736(02)00352-8 11997125

[B42] Msanne J. Xu D. Konda A. R. Casas-Mollano J. A. Awada T. Cahoon E. B. (2012). Metabolic and gene expression changes triggered by nitrogen deprivation in the photoautotrophically grown microalgae *Chlamydomonas reinhardtii* and *Coccomyxa* sp. C-169. *Phytochemistry* 75 50–59. 10.1016/j.phytochem.2011.12.007 22226037

[B43] Pal D. Khozin-Goldberg I. Cohen Z. Boussiba S. (2011). The effect of light, salinity, and nitrogen availability on lipid production by *Nannochloropsis* sp. *Appl. Microbiol. Biotechnol.* 90 1429–1441. 10.1007/s00253-011-3170-1 21431397

[B44] Pruvost J. Van Vooren G. Cogne G. Legrand J. (2009). Investigation of biomass and lipids production with Neochloris oleoabundans in photobioreactor. *Bioresour. Technol.* 100 5988–5995. 10.1016/j.biortech.2009.06.004 19560349

[B45] Radakovits R. Jinkerson R. E. Darzins A. Posewitz M. C. (2010). Genetic engineering of algae for enhanced biofuel production. *Eukaryot. Cell* 9 486–501. 10.1128/EC.00364-09 20139239 PMC2863401

[B46] Ras M. Steyer J.-P. Bernard O. (2013). Temperature effect on microalgae: a crucial factor for outdoor production. *Rev. Environ. Sci. Bio/Technol.* 12 153–164. 10.1007/s11157-013-9310-6

[B47] Renaud S. M. Thinh L. Van, Lambrinidis G. Parry D. L. (2002). Effect of temperature on growth, chemical composition and fatty acid composition of tropical Australian microalgae grown in batch cultures. *Aquaculture* 211 195–214. 10.1016/S0044-8486(01)00875-4

[B48] Rodolfi L. Zittelli G. C. Bassi N. Padovani G. Biondi N. Bonini G. (2009). Microalgae for oil: Strain selection, induction of lipid synthesis and outdoor mass cultivation in a low-cost photobioreactor. *Biotechnol. Bioeng.* 102 100–112. 10.1002/bit.22033 18683258

[B49] Schmollinger S. Muhlhaus T. Boyle N. R. Blaby I. K. Casero D. Mettler T. (2014). Nitrogen-Sparing Mechanisms in *Chlamydomonas* Affect the Transcriptome, the Proteome, and Photosynthetic Metabolism. *Plant Cell* 26 1410–1435. 10.1105/tpc.113.122523 24748044 PMC4036562

[B50] Schulze S. C. P. Hulatt C. Morales-Sánchez D. Wijffels R. Kiron V. (2019). Fatty acids and proteins from cold adapted microalgae for biotechnology. *Algal Res.* 42:101604 10.1016/j.algal.2019.101604

[B51] Siaut M. Cuiné S. Cagnon C. Fessler B. Nguyen M. Carrier P. (2011). Oil accumulation in the model green alga *Chlamydomonas reinhardtii*: Characterization, variability between common laboratory strains and relationship with starch reserves. *BMC Biotechnol.* 11:7. 10.1186/1472-6750-11-7 21255402 PMC3036615

[B52] Spolaore P. Joannis-Cassan C. Duran E. Isambert A. (2006). Commercial applications of microalgae. *J. Biosci. Bioeng.* 101 87–96. 10.1263/JBB.101.87 16569602

[B53] Suzuki H. Hulatt C. J. Wijffels R. H. Kiron V. (2018). Growth and LC-PUFA production of the cold-adapted microalga *Koliella antarctica* in photobioreactors. *J. Appl. Phycol.* 31 981–997. 10.1007/s10811-018-1606-z

[B54] Takeshita T. Ota S. Yamazaki T. Hirata A. Zachleder V. Kawano S. (2014). Starch and lipid accumulation in eight strains of six *Chlorella* species under comparatively high light intensity and aeration culture conditions. *Bioresour. Technol.* 158 127–134. 10.1016/j.biortech.2014.01.135 24583913

[B55] Thompson A. R. (1950). A colorimetric method for the determination of esters. *Aust. J. Chem.* 3 128–135. 10.1071/CH9500128

[B56] Tiwari A. Kiran T. (2018). Biofuels from Microalgae. *Adv. Biofuels Bioenergy* 2018:73012 10.5772/intechopen.73012

[B57] Work V. H. Radakovits R. Jinkerson R. E. Meuser J. E. Elliott L. G. Vinyard D. J. (2010). Increased lipid accumulation in the Chlamydomonas reinhardtii sta7-10 starchless isoamylase mutant and increased carbohydrate synthesis in complemented strains. *Eukaryot. Cell* 9 1251–1261. 10.1128/EC.00075-10 20562225 PMC2918938

[B58] Xin L. Hong-ying H. Yu-ping Z. (2011). Growth and lipid accumulation properties of a freshwater microalga *Scenedesmus* sp. under different cultivation temperature. *Bioresour. Technol.* 102 3098–3102. 10.1016/j.biortech.2010.10.055 21055924

[B59] Xin L. Hong-ying H. Ke G. Ying-xue S. (2010). Effects of different nitrogen and phosphorus concentrations on the growth, nutrient uptake, and lipid accumulation of a freshwater microalga *Scenedesmus* sp. *Bioresour. Technol.* 101 5494–5500. 10.1016/j.biortech.2010.02.016 20202827

[B60] Zhu L. D. Li Z. H. Hiltunen E. (2016). Strategies for Lipid Production Improvement in Microalgae as a Biodiesel Feedstock. *Biomed Res. Int.* 2016:8792548. 10.1155/2016/8792548 27725942 PMC5048031

