# Supplementary material for: Temperature-Dependent Lipid Accumulation in the Polar Marine Microalga Chlamydomonas malina RCC2488
Source: Front Plant Sci. 2020 Dec 23;11:619064. doi: 10.3389/fpls.2020.619064 (PMC7785989; doi:10.3389/fpls.2020.619064)
Supplement: Supplementary file 1 [file Table_1.docx]

Temperature-dependent lipid accumulation in the polar marine microalga *Chlamydomonas malina* RCC2488

Daniela Morales-Sánchez ^1,2*^, Peter S. C. Schulze ^2,3^, Viswanath Kiron^2^, Rene H. Wijffels ^2,4^

^1^The Norwegian College of Fishery Science, Faculty of Biosciences, Fisheries and Economics, UiT – The Arctic University of Norway, Tromsø, Norway

^2^Faculty of Biosciences and Aquaculture, Nord University, Bodø, Norway

^3^Green Colab – Associação Oceano Verde, University of Algarve, Campus de Gambelas, Faro, Portugal.

^4^Bioprocess Engineering, AlgaePARC, Wageningen University, Wageningen, Netherlands

***Correspondence:**Daniela Morales-Sánchez

[daniela.morales-sanchez@uit.no](mailto:daniela.morales-sanchez@uit.no)

**Statistical Procedures**

**A = 4 °C B = 8 °C C = 15 °C**

**Productivities at +N**

| Data sets analyzed | A-C |
| --- | --- |
|  |  |
| ANOVA summary |  |
| F | 602.2 |
| P value | <0.0001 |
| P value summary | **** |
| Significant diff. among means (P < 0.05)? | Yes |
| R square | 0.995 |
|  |  |
| Brown-Forsythe test |  |
| F (DFn, DFd) | 0.5850 (2, 6) |
| P value | 0.586 |
| P value summary | ns |
| Are SDs significantly different (P < 0.05)? | No |

| ANOVA table | SS | DF | MS | F (DFn, DFd) | P value |
| --- | --- | --- | --- | --- | --- |
| Treatment (between columns) | 506900 | 2 | 253450 | F (2, 6) = 602.2 | P<0.0001 |
| Residual (within columns) | 2525 | 6 | 420.9 |  |  |
| Total | 509425 | 8 |  |  |  |

| Number of families | 1 |
| --- | --- |
| Number of comparisons per family | 3 |
| Alpha | 0.05 |

| Tukey's multiple comparisons test | Mean Diff. | 95.00% CI of diff. | Significant? | Summary | Adjusted P Value | |
| --- | --- | --- | --- | --- | --- | --- |
| Column A vs. Column B | 129.3 | 77.87 to 180.7 | Yes | *** | 0.0006 | A-B |
| Column A vs. Column C | 555.5 | 504.1 to 606.9 | Yes | **** | <0.0001 | A-C |
| Column B vs. Column C | 426.2 | 374.8 to 477.6 | Yes | **** | <0.0001 | B-C |

| Test details | Mean 1 | Mean 2 | Mean Diff. | SE of diff. | n1 | n2 | q | DF |
| --- | --- | --- | --- | --- | --- | --- | --- | --- |
| Column A vs. Column B | 701.9 | 572.6 | 129.3 | 16.75 | 3 | 3 | 10.91 | 6 |
| Column A vs. Column C | 701.9 | 146.4 | 555.5 | 16.75 | 3 | 3 | 46.9 | 6 |
| Column B vs. Column C | 572.6 | 146.4 | 426.2 | 16.75 | 3 | 3 | 35.98 | 6 |

**Productivities at -N**

| Data sets analyzed | A-C |
| --- | --- |
| ANOVA summary |  |
| F | 580.3 |
| P value | <0.0001 |
| P value summary | **** |
| Significant diff. among means (P < 0.05)? | Yes |
| R square | 0.9949 |
|  |  |
| Brown-Forsythe test |  |
| F (DFn, DFd) | 0.6358 (2, 6) |
| P value | 0.5618 |
| P value summary | ns |
| Are SDs significantly different (P < 0.05)? | No |

| ANOVA table | SS | DF | MS | F (DFn, DFd) | P value |
| --- | --- | --- | --- | --- | --- |
| Treatment (between columns) | 386416 | 2 | 193208 | F (2, 6) = 580.3 | P<0.0001 |
| Residual (within columns) | 1998 | 6 | 332.9 |  |  |
| Total | 388414 | 8 |  |  |  |

| Number of families | 1 |
| --- | --- |
| Number of comparisons per family | 3 |
| Alpha | 0.05 |

| Tukey's multiple comparisons test | Mean Diff. | 95.00% CI of diff. | Significant? | Summary | Adjusted P Value | |
| --- | --- | --- | --- | --- | --- | --- |
| 4 vs. 8 | 168.8 | 123.1 to 214.5 | Yes | **** | <0.0001 | A-B |
| 4 vs. 15 | 498.9 | 453.2 to 544.6 | Yes | **** | <0.0001 | A-C |
| 8 vs. 15 | 330.1 | 284.4 to 375.8 | Yes | **** | <0.0001 | B-C |

| Test details | Mean 1 | Mean 2 | Mean Diff. | SE of diff. | n1 | n2 | q | DF |
| --- | --- | --- | --- | --- | --- | --- | --- | --- |
| 4 vs. 8 | 570.3 | 401.5 | 168.8 | 14.9 | 3 | 3 | 16.02 | 6 |
| 4 vs. 15 | 570.3 | 71.4 | 498.9 | 14.9 | 3 | 3 | 47.36 | 6 |
| 8 vs. 15 | 401.5 | 71.4 | 330.1 | 14.9 | 3 | 3 | 31.34 | 6 |

**Protein +N**

| Data sets analyzed | A-C |
| --- | --- |
|  |  |
| ANOVA summary |  |
| F | 86.24 |
| P value | <0.0001 |
| P value summary | **** |
| Significant diff. among means (P < 0.05)? | Yes |
| R square | 0.9664 |
|  |  |
| Brown-Forsythe test |  |
| F (DFn, DFd) | 0.5285 (2, 6) |
| P value | 0.6146 |
| P value summary | ns |
| Are SDs significantly different (P < 0.05)? | No |

| ANOVA table | SS | DF | MS | F (DFn, DFd) | P value |
| --- | --- | --- | --- | --- | --- |
| Treatment (between columns) | 0.04327 | 2 | 0.02164 | F (2, 6) = 86.24 | P<0.0001 |
| Residual (within columns) | 0.001505 | 6 | 0.0002509 |  |  |
| Total | 0.04478 | 8 |  |  |  |

| Number of families | 1 |
| --- | --- |
| Number of comparisons per family | 3 |
| Alpha | 0.05 |

| Tukey's multiple comparisons test | Mean Diff. | 95.00% CI of diff. | Significant? | Summary | Adjusted P Value |  |
| --- | --- | --- | --- | --- | --- | --- |
| 4 vs. 8 | 0.1357 | 0.09599 to 0.1753 | Yes | *** | 0.0001 | A-B |
| 4 vs. 15 | 0.1563 | 0.1167 to 0.1960 | Yes | **** | <0.0001 | A-C |
| 8 vs. 15 | 0.02067 | -0.01901 to 0.06035 | No | ns | 0.3167 | B-C |

| Test details | Mean 1 | Mean 2 | Mean Diff. | SE of diff. | n1 | n2 | q | DF |
| --- | --- | --- | --- | --- | --- | --- | --- | --- |
| 4 vs. 8 | 0.4117 | 0.276 | 0.1357 | 0.01293 | 3 | 3 | 14.84 | 6 |
| 4 vs. 15 | 0.4117 | 0.2553 | 0.1563 | 0.01293 | 3 | 3 | 17.1 | 6 |
| 8 vs. 15 | 0.276 | 0.2553 | 0.02067 | 0.01293 | 3 | 3 | 2.26 | 6 |

**Protein -N**

| Data sets analyzed | A-C |
| --- | --- |
|  |  |
| ANOVA summary |  |
| F | 0.3937 |
| P value | 0.6908 |
| P value summary | ns |
| Significant diff. among means (P < 0.05)? | No |
| R square | 0.116 |
|  |  |
| Brown-Forsythe test |  |
| F (DFn, DFd) | 0.3626 (2, 6) |
| P value | 0.7102 |
| P value summary | ns |
| Are SDs significantly different (P < 0.05)? | No |

| ANOVA table | SS | DF | MS | F (DFn, DFd) | P value |
| --- | --- | --- | --- | --- | --- |
| Treatment (between columns) | 0.0006819 | 2 | 0.0003409 | F (2, 6) = 0.3937 | P=0.6908 |
| Residual (within columns) | 0.005195 | 6 | 0.0008659 |  |  |
| Total | 0.005877 | 8 |  |  |  |

| Number of families | 1 |
| --- | --- |
| Number of comparisons per family | 3 |
| Alpha | 0.05 |

| Tukey's multiple comparisons test | Mean Diff. | 95.00% CI of diff. | Significant? | Summary | Adjusted P Value |  |
| --- | --- | --- | --- | --- | --- | --- |
| 4 vs. 8 | -0.0062 | -0.07992 to 0.06752 | No | ns | 0.9642 | A-B |
| 4 vs. 15 | -0.02077 | -0.09449 to 0.05295 | No | ns | 0.6803 | A-C |
| 8 vs. 15 | -0.01457 | -0.08829 to 0.05915 | No | ns | 0.8221 | B-C |

| Test details | Mean 1 | Mean 2 | Mean Diff. | SE of diff. | n1 | n2 | q | DF |
| --- | --- | --- | --- | --- | --- | --- | --- | --- |
| 4 vs. 8 | 0.1523 | 0.1585 | -0.0062 | 0.02403 | 3 | 3 | 0.3649 | 6 |
| 4 vs. 15 | 0.1523 | 0.1731 | -0.02077 | 0.02403 | 3 | 3 | 1.222 | 6 |
| 8 vs. 15 | 0.1585 | 0.1731 | -0.01457 | 0.02403 | 3 | 3 | 0.8574 | 6 |

**Carbohydrates +N**

| Data sets analyzed | A-C |
| --- | --- |
|  |  |
| ANOVA summary |  |
| F | 1.29 |
| P value | 0.342 |
| P value summary | ns |
| Significant diff. among means (P < 0.05)? | No |
| R square | 0.3007 |
|  |  |
| Brown-Forsythe test |  |
| F (DFn, DFd) | 0.6761 (2, 6) |
| P value | 0.5435 |
| P value summary | ns |
| Are SDs significantly different (P < 0.05)? | No |

| ANOVA table | SS | DF | MS | F (DFn, DFd) | P value |
| --- | --- | --- | --- | --- | --- |
| Treatment (between columns) | 0.001591 | 2 | 0.0007956 | F (2, 6) = 1.290 | P=0.3420 |
| Residual (within columns) | 0.0037 | 6 | 0.0006167 |  |  |
| Total | 0.005292 | 8 |  |  |  |

| Number of families | 1 |
| --- | --- |
| Number of comparisons per family | 3 |
| Alpha | 0.05 |

| Tukey's multiple comparisons test | Mean Diff. | 95.00% CI of diff. | Significant? | Summary | Adjusted P Value |  |
| --- | --- | --- | --- | --- | --- | --- |
| 4 vs. 8 | -0.0188 | -0.08101 to 0.04341 | No | ns | 0.6446 | A-B |
| 4 vs. 15 | -0.03243 | -0.09465 to 0.02978 | No | ns | 0.3161 | A-C |
| 8 vs. 15 | -0.01363 | -0.07585 to 0.04858 | No | ns | 0.7873 | B-C |

| Test details | Mean 1 | Mean 2 | Mean Diff. | SE of diff. | n1 | n2 | q | DF |
| --- | --- | --- | --- | --- | --- | --- | --- | --- |
| 4 vs. 8 | 0.2264 | 0.2452 | -0.0188 | 0.02028 | 3 | 3 | 1.311 | 6 |
| 4 vs. 15 | 0.2264 | 0.2589 | -0.03243 | 0.02028 | 3 | 3 | 2.262 | 6 |
| 8 vs. 15 | 0.2452 | 0.2589 | -0.01363 | 0.02028 | 3 | 3 | 0.9509 | 6 |

**Carbohydrates -N**

| Data sets analyzed | A-C |
| --- | --- |
|  |  |
| ANOVA summary |  |
| F | 24.1 |
| P value | 0.0014 |
| P value summary | ** |
| Significant diff. among means (P < 0.05)? | Yes |
| R square | 0.8893 |
|  |  |
| Brown-Forsythe test |  |
| F (DFn, DFd) | 0.1719 (2, 6) |
| P value | 0.846 |
| P value summary | ns |
| Are SDs significantly different (P < 0.05)? | No |

| ANOVA table | SS | DF | MS | F (DFn, DFd) | P value |
| --- | --- | --- | --- | --- | --- |
| Treatment (between columns) | 0.02585 | 2 | 0.01293 | F (2, 6) = 24.10 | P=0.0014 |
| Residual (within columns) | 0.003219 | 6 | 0.0005365 |  |  |
| Total | 0.02907 | 8 |  |  |  |

| Number of families | 1 |
| --- | --- |
| Number of comparisons per family | 3 |
| Alpha | 0.05 |

| Tukey's multiple comparisons test | Mean Diff. | 95.00% CI of diff. | Significant? | Summary | Adjusted P Value |  |
| --- | --- | --- | --- | --- | --- | --- |
| 4 vs. 8 | -0.04003 | -0.09806 to 0.01799 | No | ns | 0.1664 | A-B |
| 4 vs. 15 | 0.08827 | 0.03024 to 0.1463 | Yes | ** | 0.0082 | A-C |
| 8 vs. 15 | 0.1283 | 0.07027 to 0.1863 | Yes | ** | 0.0012 | B-C |

| Test details | Mean 1 | Mean 2 | Mean Diff. | SE of diff. | n1 | n2 | q | DF |
| --- | --- | --- | --- | --- | --- | --- | --- | --- |
| 4 vs. 8 | 0.4049 | 0.4449 | -0.04003 | 0.01891 | 3 | 3 | 2.994 | 6 |
| 4 vs. 15 | 0.4049 | 0.3166 | 0.08827 | 0.01891 | 3 | 3 | 6.6 | 6 |
| 8 vs. 15 | 0.4449 | 0.3166 | 0.1283 | 0.01891 | 3 | 3 | 9.594 | 6 |

**Lipids +N**

| Data sets analyzed | A-C |
| --- | --- |
|  |  |
| ANOVA summary |  |
| F | 29.68 |
| P value | 0.0008 |
| P value summary | *** |
| Significant diff. among means (P < 0.05)? | Yes |
| R square | 0.9082 |
|  |  |
| Brown-Forsythe test |  |
| F (DFn, DFd) | 1.796 (2, 6) |
| P value | 0.2448 |
| P value summary | ns |
| Are SDs significantly different (P < 0.05)? | No |

| ANOVA table | SS | DF | MS | F (DFn, DFd) | P value |
| --- | --- | --- | --- | --- | --- |
| Treatment (between columns) | 0.02107 | 2 | 0.01054 | F (2, 6) = 29.68 | P=0.0008 |
| Residual (within columns) | 0.00213 | 6 | 0.000355 |  |  |
| Total | 0.0232 | 8 |  |  |  |

| Number of families | 1 |
| --- | --- |
| Number of comparisons per family | 3 |
| Alpha | 0.05 |

| Tukey's multiple comparisons test | Mean Diff. | 95.00% CI of diff. | Significant? | Summary | Adjusted P Value |  |
| --- | --- | --- | --- | --- | --- | --- |
| 4 vs. 8 | -0.1113 | -0.1585 to -0.06407 | Yes | *** | 0.0009 | A-B |
| 4 vs. 15 | -0.091 | -0.1382 to -0.04380 | Yes | ** | 0.0025 | A-C |
| 8 vs. 15 | 0.02027 | -0.02693 to 0.06747 | No | ns | 0.437 | B-C |

| Test details | Mean 1 | Mean 2 | Mean Diff. | SE of diff. | n1 | n2 | q | DF |
| --- | --- | --- | --- | --- | --- | --- | --- | --- |
| 4 vs. 8 | 0.2017 | 0.3129 | -0.1113 | 0.01538 | 3 | 3 | 10.23 | 6 |
| 4 vs. 15 | 0.2017 | 0.2927 | -0.091 | 0.01538 | 3 | 3 | 8.366 | 6 |
| 8 vs. 15 | 0.3129 | 0.2927 | 0.02027 | 0.01538 | 3 | 3 | 1.863 | 6 |

**Lipids -N**

| Data sets analyzed | A-C |
| --- | --- |
|  |  |
| ANOVA summary |  |
| F | 19.38 |
| P value | 0.0024 |
| P value summary | ** |
| Significant diff. among means (P < 0.05)? | Yes |
| R square | 0.8659 |
|  |  |
| Brown-Forsythe test |  |
| F (DFn, DFd) | 0.3476 (2, 6) |
| P value | 0.7198 |
| P value summary | ns |
| Are SDs significantly different (P < 0.05)? | No |

| ANOVA table | SS | DF | MS | F (DFn, DFd) | P value |
| --- | --- | --- | --- | --- | --- |
| Treatment (between columns) | 0.01311 | 2 | 0.006555 | F (2, 6) = 19.38 | P=0.0024 |
| Residual (within columns) | 0.00203 | 6 | 0.0003383 |  |  |
| Total | 0.01514 | 8 |  |  |  |

| Number of families | 1 |
| --- | --- |
| Number of comparisons per family | 3 |
| Alpha | 0.05 |

| Tukey's multiple comparisons test | Mean Diff. | 95.00% CI of diff. | Significant? | Summary | Adjusted P Value |  |
| --- | --- | --- | --- | --- | --- | --- |
| 4 vs. 8 | 0.007533 | -0.03855 to 0.05361 | No | ns | 0.8733 | A-B |
| 4 vs. 15 | -0.07693 | -0.1230 to -0.03085 | Yes | ** | 0.0052 | A-C |
| 8 vs. 15 | -0.08447 | -0.1305 to -0.03839 | Yes | ** | 0.0033 | B-C |

| Test details | Mean 1 | Mean 2 | Mean Diff. | SE of diff. | n1 | n2 | q | DF |
| --- | --- | --- | --- | --- | --- | --- | --- | --- |
| 4 vs. 8 | 0.3218 | 0.3143 | 0.007533 | 0.01502 | 3 | 3 | 0.7094 | 6 |
| 4 vs. 15 | 0.3218 | 0.3987 | -0.07693 | 0.01502 | 3 | 3 | 7.245 | 6 |
| 8 vs. 15 | 0.3143 | 0.3987 | -0.08447 | 0.01502 | 3 | 3 | 7.954 | 6 |

**SFA in polar fraction at +N**

| Data sets analyzed | A-C |
| --- | --- |
|  |  |
| ANOVA summary |  |
| F | 44 |
| P value | 0.0003 |
| P value summary | *** |
| Significant diff. among means (P < 0.05)? | Yes |
| R square | 0.9362 |
|  |  |
| Brown-Forsythe test |  |
| F (DFn, DFd) | 0.2045 (2, 6) |
| P value | 0.8205 |
| P value summary | ns |
| Are SDs significantly different (P < 0.05)? | No |

| ANOVA table | SS | DF | MS | F (DFn, DFd) | P value |
| --- | --- | --- | --- | --- | --- |
| Treatment (between columns) | 821.5 | 2 | 410.8 | F (2, 6) = 44.00 | P=0.0003 |
| Residual (within columns) | 56.01 | 6 | 9.334 |  |  |
| Total | 877.5 | 8 |  |  |  |

| Number of families | 1 |
| --- | --- |
| Number of comparisons per family | 3 |
| Alpha | 0.05 |

| Tukey's multiple comparisons test | Mean Diff. | 95.00% CI of diff. | Significant? | Summary | Adjusted P Value |  |
| --- | --- | --- | --- | --- | --- | --- |
| 4 vs. 8 | 0.1333 | -7.521 to 7.787 | No | ns | 0.9984 | A-B |
| 4 vs. 15 | -20.2 | -27.85 to -12.55 | Yes | *** | 0.0005 | A-C |
| 8 vs. 15 | -20.33 | -27.99 to -12.68 | Yes | *** | 0.0004 | B-C |

| Test details | Mean 1 | Mean 2 | Mean Diff. | SE of diff. | n1 | n2 | q | DF |
| --- | --- | --- | --- | --- | --- | --- | --- | --- |
| 4 vs. 8 | 14.2 | 14.07 | 0.1333 | 2.495 | 3 | 3 | 0.07559 | 6 |
| 4 vs. 15 | 14.2 | 34.4 | -20.2 | 2.495 | 3 | 3 | 11.45 | 6 |
| 8 vs. 15 | 14.07 | 34.4 | -20.33 | 2.495 | 3 | 3 | 11.53 | 6 |

**SFA in TAG fraction at +N**

| Data sets analyzed | A-C |
| --- | --- |
|  |  |
| ANOVA summary |  |
| F | 0.5043 |
| P value | 0.6274 |
| P value summary | ns |
| Significant diff. among means (P < 0.05)? | No |
| R square | 0.1439 |
|  |  |
| Brown-Forsythe test |  |
| F (DFn, DFd) | 0.01529 (2, 6) |
| P value | 0.9849 |
| P value summary | ns |
| Are SDs significantly different (P < 0.05)? | No |

| ANOVA table | SS | DF | MS | F (DFn, DFd) | P value |
| --- | --- | --- | --- | --- | --- |
| Treatment (between columns) | 32.28 | 2 | 16.14 | F (2, 6) = 0.5043 | P=0.6274 |
| Residual (within columns) | 192 | 6 | 32.01 |  |  |
| Total | 224.3 | 8 |  |  |  |

| Number of families | 1 |
| --- | --- |
| Number of comparisons per family | 3 |
| Alpha | 0.05 |

| Tukey's multiple comparisons test | Mean Diff. | 95.00% CI of diff. | Significant? | Summary | Adjusted P Value |  |
| --- | --- | --- | --- | --- | --- | --- |
| 4 vs. 8 | -4.067 | -18.24 to 10.11 | No | ns | 0.6711 | A-B |
| 4 vs. 15 | -0.1 | -14.27 to 14.07 | No | ns | 0.9997 | A-C |
| 8 vs. 15 | 3.967 | -10.21 to 18.14 | No | ns | 0.6834 | B-C |

| Test details | Mean 1 | Mean 2 | Mean Diff. | SE of diff. | n1 | n2 | q | DF |
| --- | --- | --- | --- | --- | --- | --- | --- | --- |
| 4 vs. 8 | 25.8 | 29.87 | -4.067 | 4.619 | 3 | 3 | 1.245 | 6 |
| 4 vs. 15 | 25.8 | 25.9 | -0.1 | 4.619 | 3 | 3 | 0.03061 | 6 |
| 8 vs. 15 | 29.87 | 25.9 | 3.967 | 4.619 | 3 | 3 | 1.214 | 6 |

**MUFA in polar fraction at +N**

| Data sets analyzed | A-C |
| --- | --- |
|  |  |
| ANOVA summary |  |
| F | 358.4 |
| P value | <0.0001 |
| P value summary | **** |
| Significant diff. among means (P < 0.05)? | Yes |
| R square | 0.9917 |
|  |  |
| Brown-Forsythe test |  |
| F (DFn, DFd) | 1.917 (2, 6) |
| P value | 0.2271 |
| P value summary | ns |
| Are SDs significantly different (P < 0.05)? | No |

| ANOVA table | SS | DF | MS | F (DFn, DFd) | P value |
| --- | --- | --- | --- | --- | --- |
| Treatment (between columns) | 21256 | 2 | 10628 | F (2, 6) = 358.4 | P<0.0001 |
| Residual (within columns) | 177.9 | 6 | 29.66 |  |  |
| Total | 21433 | 8 |  |  |  |

| Number of families | 1 |
| --- | --- |
| Number of comparisons per family | 3 |
| Alpha | 0.05 |

| Tukey's multiple comparisons test | Mean Diff. | 95.00% CI of diff. | Significant? | Summary | Adjusted P Value |  |
| --- | --- | --- | --- | --- | --- | --- |
| 4 vs. 8 | 105.5 | 91.86 to 119.1 | Yes | **** | <0.0001 | A-B |
| 4 vs. 15 | 100.5 | 86.86 to 114.1 | Yes | **** | <0.0001 | A-C |
| 8 vs. 15 | -5 | -18.64 to 8.643 | No | ns | 0.5351 | B-C |

| Test details | Mean 1 | Mean 2 | Mean Diff. | SE of diff. | n1 | n2 | q | DF |
| --- | --- | --- | --- | --- | --- | --- | --- | --- |
| 4 vs. 8 | 115.5 | 10 | 105.5 | 4.446 | 3 | 3 | 33.55 | 6 |
| 4 vs. 15 | 115.5 | 15 | 100.5 | 4.446 | 3 | 3 | 31.96 | 6 |
| 8 vs. 15 | 10 | 15 | -5 | 4.446 | 3 | 3 | 1.59 | 6 |

**MUFA in TAG fraction +N**

| Data sets analyzed | A-C |
| --- | --- |
|  |  |
| ANOVA summary |  |
| F | 167.6 |
| P value | <0.0001 |
| P value summary | **** |
| Significant diff. among means (P < 0.05)? | Yes |
| R square | 0.9824 |
|  |  |
| Brown-Forsythe test |  |
| F (DFn, DFd) | 1.503 (2, 6) |
| P value | 0.2957 |
| P value summary | ns |
| Are SDs significantly different (P < 0.05)? | No |

| ANOVA table | SS | DF | MS | F (DFn, DFd) | P value |
| --- | --- | --- | --- | --- | --- |
| Treatment (between columns) | 16570 | 2 | 8285 | F (2, 6) = 167.6 | P<0.0001 |
| Residual (within columns) | 296.6 | 6 | 49.44 |  |  |
| Total | 16867 | 8 |  |  |  |

| Number of families | 1 |
| --- | --- |
| Number of comparisons per family | 3 |
| Alpha | 0.05 |

| Tukey's multiple comparisons test | Mean Diff. | 95.00% CI of diff. | Significant? | Summary | Adjusted P Value |  |
| --- | --- | --- | --- | --- | --- | --- |
| 4 vs. 8 | -68.17 | -85.78 to -50.55 | Yes | **** | <0.0001 | A-B |
| 4 vs. 15 | -103.4 | -121.0 to -85.75 | Yes | **** | <0.0001 | A-C |
| 8 vs. 15 | -35.2 | -52.82 to -17.58 | Yes | ** | 0.0021 | B-C |

| Test details | Mean 1 | Mean 2 | Mean Diff. | SE of diff. | n1 | n2 | q | DF |
| --- | --- | --- | --- | --- | --- | --- | --- | --- |
| 4 vs. 8 | 11.43 | 79.6 | -68.17 | 5.741 | 3 | 3 | 16.79 | 6 |
| 4 vs. 15 | 11.43 | 114.8 | -103.4 | 5.741 | 3 | 3 | 25.46 | 6 |
| 8 vs. 15 | 79.6 | 114.8 | -35.2 | 5.741 | 3 | 3 | 8.671 | 6 |

**PUFA in polar +N**

| Data sets analyzed | A-C |
| --- | --- |
|  |  |
| ANOVA summary |  |
| F | 506.3 |
| P value | <0.0001 |
| P value summary | **** |
| Significant diff. among means (P < 0.05)? | Yes |
| R square | 0.9941 |
|  |  |
| Brown-Forsythe test |  |
| F (DFn, DFd) | 1.210 (2, 6) |
| P value | 0.3619 |
| P value summary | ns |
| Are SDs significantly different (P < 0.05)? | No |

| ANOVA table | SS | DF | MS | F (DFn, DFd) | P value |
| --- | --- | --- | --- | --- | --- |
| Treatment (between columns) | 20815 | 2 | 10407 | F (2, 6) = 506.3 | P<0.0001 |
| Residual (within columns) | 123.3 | 6 | 20.56 |  |  |
| Total | 20938 | 8 |  |  |  |

| Number of families | 1 |
| --- | --- |
| Number of comparisons per family | 3 |
| Alpha | 0.05 |

| Tukey's multiple comparisons test | Mean Diff. | 95.00% CI of diff. | Significant? | Summary | Adjusted P Value |  |
| --- | --- | --- | --- | --- | --- | --- |
| 4 vs. 8 | 86.5 | 75.14 to 97.86 | Yes | **** | <0.0001 | A-B |
| 4 vs. 15 | 112.5 | 101.1 to 123.9 | Yes | **** | <0.0001 | A-C |
| 8 vs. 15 | 26 | 14.64 to 37.36 | Yes | ** | 0.001 | B-C |

| Test details | Mean 1 | Mean 2 | Mean Diff. | SE of diff. | n1 | n2 | q | DF |
| --- | --- | --- | --- | --- | --- | --- | --- | --- |
| 4 vs. 8 | 122.5 | 36 | 86.5 | 3.702 | 3 | 3 | 33.04 | 6 |
| 4 vs. 15 | 122.5 | 10 | 112.5 | 3.702 | 3 | 3 | 42.98 | 6 |
| 8 vs. 15 | 36 | 10 | 26 | 3.702 | 3 | 3 | 9.932 | 6 |

**PUFA in TAG at +N**

| Data sets analyzed | A-C |
| --- | --- |
|  |  |
| ANOVA summary |  |
| F | 240.2 |
| P value | <0.0001 |
| P value summary | **** |
| Significant diff. among means (P < 0.05)? | Yes |
| R square | 0.9877 |
|  |  |
| Brown-Forsythe test |  |
| F (DFn, DFd) | 0.08396 (2, 6) |
| P value | 0.9205 |
| P value summary | ns |
| Are SDs significantly different (P < 0.05)? | No |

| ANOVA table | SS | DF | MS | F (DFn, DFd) | P value |
| --- | --- | --- | --- | --- | --- |
| Treatment (between columns) | 11000 | 2 | 5500 | F (2, 6) = 240.2 | P<0.0001 |
| Residual (within columns) | 137.4 | 6 | 22.9 |  |  |
| Total | 11137 | 8 |  |  |  |

| Number of families | 1 |
| --- | --- |
| Number of comparisons per family | 3 |
| Alpha | 0.05 |

| Tukey's multiple comparisons test | Mean Diff. | 95.00% CI of diff. | Significant? | Summary | Adjusted P Value |  |
| --- | --- | --- | --- | --- | --- | --- |
| 4 vs. 8 | -85.63 | -97.62 to -73.65 | Yes | **** | <0.0001 | A-B |
| 4 vs. 15 | -43 | -54.99 to -31.01 | Yes | **** | <0.0001 | A-C |
| 8 vs. 15 | 42.63 | 30.65 to 54.62 | Yes | **** | <0.0001 | B-C |

| Test details | Mean 1 | Mean 2 | Mean Diff. | SE of diff. | n1 | n2 | q | DF |
| --- | --- | --- | --- | --- | --- | --- | --- | --- |
| 4 vs. 8 | 36.87 | 122.5 | -85.63 | 3.907 | 3 | 3 | 31 | 6 |
| 4 vs. 15 | 36.87 | 79.87 | -43 | 3.907 | 3 | 3 | 15.57 | 6 |
| 8 vs. 15 | 122.5 | 79.87 | 42.63 | 3.907 | 3 | 3 | 15.43 | 6 |

**Total polar lipids at +N**

| Data sets analyzed | A-C |
| --- | --- |
|  |  |
| ANOVA summary |  |
| F | 290 |
| P value | <0.0001 |
| P value summary | **** |
| Significant diff. among means (P < 0.05)? | Yes |
| R square | 0.9898 |
|  |  |
| Brown-Forsythe test |  |
| F (DFn, DFd) | 1.305 (2, 6) |
| P value | 0.3384 |
| P value summary | ns |
| Are SDs significantly different (P < 0.05)? | No |

| ANOVA table | SS | DF | MS | F (DFn, DFd) | P value |
| --- | --- | --- | --- | --- | --- |
| Treatment (between columns) | 74088 | 2 | 37044 | F (2, 6) = 290.0 | P<0.0001 |
| Residual (within columns) | 766.3 | 6 | 127.7 |  |  |
| Total | 74854 | 8 |  |  |  |

| Number of families | 1 |
| --- | --- |
| Number of comparisons per family | 3 |
| Alpha | 0.05 |

| Tukey's multiple comparisons test | Mean Diff. | 95.00% CI of diff. | Significant? | Summary | Adjusted P Value |  |
| --- | --- | --- | --- | --- | --- | --- |
| 4 vs. 8 | 192.1 | 163.8 to 220.4 | Yes | **** | <0.0001 | A-B |
| 4 vs. 15 | 192.8 | 164.5 to 221.1 | Yes | **** | <0.0001 | A-C |
| 8 vs. 15 | 0.6667 | -27.65 to 28.98 | No | ns | 0.9971 | B-C |

| Test details | Mean 1 | Mean 2 | Mean Diff. | SE of diff. | n1 | n2 | q | DF |
| --- | --- | --- | --- | --- | --- | --- | --- | --- |
| 4 vs. 8 | 252.2 | 60.07 | 192.1 | 9.228 | 3 | 3 | 29.45 | 6 |
| 4 vs. 15 | 252.2 | 59.4 | 192.8 | 9.228 | 3 | 3 | 29.55 | 6 |
| 8 vs. 15 | 60.07 | 59.4 | 0.6667 | 9.228 | 3 | 3 | 0.1022 | 6 |

**Total TAG lipids in +N**

| Data sets analyzed | A-C |
| --- | --- |
|  |  |
| ANOVA summary |  |
| F | 81.25 |
| P value | <0.0001 |
| P value summary | **** |
| Significant diff. among means (P < 0.05)? | Yes |
| R square | 0.9644 |
|  |  |
| Brown-Forsythe test |  |
| F (DFn, DFd) | 0.3038 (2, 6) |
| P value | 0.7487 |
| P value summary | ns |
| Are SDs significantly different (P < 0.05)? | No |

| ANOVA table | SS | DF | MS | F (DFn, DFd) | P value |
| --- | --- | --- | --- | --- | --- |
| Treatment (between columns) | 46504 | 2 | 23252 | F (2, 6) = 81.25 | P<0.0001 |
| Residual (within columns) | 1717 | 6 | 286.2 |  |  |
| Total | 48222 | 8 |  |  |  |

| Number of families | 1 |
| --- | --- |
| Number of comparisons per family | 3 |
| Alpha | 0.05 |

| Tukey's multiple comparisons test | Mean Diff. | 95.00% CI of diff. | Significant? | Summary | Adjusted P Value |  |
| --- | --- | --- | --- | --- | --- | --- |
| 4 vs. 8 | -157.9 | -200.2 to -115.5 | Yes | **** | <0.0001 | A-B |
| 4 vs. 15 | -146.5 | -188.8 to -104.1 | Yes | *** | 0.0001 | A-C |
| 8 vs. 15 | 11.4 | -30.98 to 53.78 | No | ns | 0.7024 | B-C |

| Test details | Mean 1 | Mean 2 | Mean Diff. | SE of diff. | n1 | n2 | q | DF |
| --- | --- | --- | --- | --- | --- | --- | --- | --- |
| 4 vs. 8 | 74.1 | 232 | -157.9 | 13.81 | 3 | 3 | 16.16 | 6 |
| 4 vs. 15 | 74.1 | 220.6 | -146.5 | 13.81 | 3 | 3 | 15 | 6 |
| 8 vs. 15 | 232 | 220.6 | 11.4 | 13.81 | 3 | 3 | 1.167 | 6 |

**SFA in polar fraction at -N**

| Data sets analyzed | A-C |
| --- | --- |
|  |  |
| ANOVA summary |  |
| F | 6.426 |
| P value | 0.0322 |
| P value summary | * |
| Significant diff. among means (P < 0.05)? | Yes |
| R square | 0.6817 |
|  |  |
| Brown-Forsythe test |  |
| F (DFn, DFd) | 0.5804 (2, 6) |
| P value | 0.5883 |
| P value summary | ns |
| Are SDs significantly different (P < 0.05)? | No |

| ANOVA table | SS | DF | MS | F (DFn, DFd) | P value |
| --- | --- | --- | --- | --- | --- |
| Treatment (between columns) | 76.38 | 2 | 38.19 | F (2, 6) = 6.426 | P=0.0322 |
| Residual (within columns) | 35.66 | 6 | 5.943 |  |  |
| Total | 112 | 8 |  |  |  |
|  |  |  |  |  |  |
| Number of families | 1 |  |  |  |  |
| Number of comparisons per family | 3 |  |  |  |  |
| Alpha | 0.05 |  |  |  |  |

| Tukey's multiple comparisons test | Mean Diff. | 95.00% CI of diff. | Significant? | Summary | Adjusted P Value |  |
| --- | --- | --- | --- | --- | --- | --- |
| 4 vs. 8 | 2.3 | -3.808 to 8.408 | No | ns | 0.5186 | A-B |
| 4 vs. 15 | 7 | 0.8925 to 13.11 | Yes | * | 0.0291 | A-C |
| 8 vs. 15 | 4.7 | -1.408 to 10.81 | No | ns | 0.1219 | B-C |

| Test details | Mean 1 | Mean 2 | Mean Diff. | SE of diff. | n1 | n2 | q | DF |
| --- | --- | --- | --- | --- | --- | --- | --- | --- |
| 4 vs. 8 | 18.37 | 16.07 | 2.3 | 1.991 | 3 | 3 | 1.634 | 6 |
| 4 vs. 15 | 18.37 | 11.37 | 7 | 1.991 | 3 | 3 | 4.973 | 6 |
| 8 vs. 15 | 16.07 | 11.37 | 4.7 | 1.991 | 3 | 3 | 3.339 | 6 |

**SFA in TAG fraction at -N**

| Data sets analyzed | A-C |
| --- | --- |
|  |  |
| ANOVA summary |  |
| F | 127.8 |
| P value | <0.0001 |
| P value summary | **** |
| Significant diff. among means (P < 0.05)? | Yes |
| R square | 0.9771 |
|  |  |
| Brown-Forsythe test |  |
| F (DFn, DFd) | 0.2113 (2, 6) |
| P value | 0.8153 |
| P value summary | ns |
| Are SDs significantly different (P < 0.05)? | No |

| ANOVA table | SS | DF | MS | F (DFn, DFd) | P value |
| --- | --- | --- | --- | --- | --- |
| Treatment (between columns) | 4938 | 2 | 2469 | F (2, 6) = 127.8 | P<0.0001 |
| Residual (within columns) | 115.9 | 6 | 19.32 |  |  |
| Total | 5054 | 8 |  |  |  |

| Number of families | 1 |
| --- | --- |
| Number of comparisons per family | 3 |
| Alpha | 0.05 |

| Tukey's multiple comparisons test | Mean Diff. | 95.00% CI of diff. | Significant? | Summary | Adjusted P Value |  |
| --- | --- | --- | --- | --- | --- | --- |
| 4 vs. 8 | -0.8333 | -11.84 to 10.18 | No | ns | 0.9709 | A-B |
| 4 vs. 15 | -50.1 | -61.11 to -39.09 | Yes | **** | <0.0001 | A-C |
| 8 vs. 15 | -49.27 | -60.28 to -38.26 | Yes | **** | <0.0001 | B-C |

| Test details | Mean 1 | Mean 2 | Mean Diff. | SE of diff. | n1 | n2 | q | DF |
| --- | --- | --- | --- | --- | --- | --- | --- | --- |
| 4 vs. 8 | 27.67 | 28.5 | -0.8333 | 3.588 | 3 | 3 | 0.3284 | 6 |
| 4 vs. 15 | 27.67 | 77.77 | -50.1 | 3.588 | 3 | 3 | 19.74 | 6 |
| 8 vs. 15 | 28.5 | 77.77 | -49.27 | 3.588 | 3 | 3 | 19.42 | 6 |

**MUFA in polar at -N**

| Data sets analyzed | A-C |
| --- | --- |
|  |  |
| ANOVA summary |  |
| F | 151.9 |
| P value | <0.0001 |
| P value summary | **** |
| Significant diff. among means (P < 0.05)? | Yes |
| R square | 0.9806 |
|  |  |
| Brown-Forsythe test |  |
| F (DFn, DFd) | 1.113 (2, 6) |
| P value | 0.3879 |
| P value summary | ns |
| Are SDs significantly different (P < 0.05)? | No |

| ANOVA table | SS | DF | MS | F (DFn, DFd) | P value |
| --- | --- | --- | --- | --- | --- |
| Treatment (between columns) | 2687 | 2 | 1343 | F (2, 6) = 151.9 | P<0.0001 |
| Residual (within columns) | 53.06 | 6 | 8.843 |  |  |
| Total | 2740 | 8 |  |  |  |

| Number of families | 1 |
| --- | --- |
| Number of comparisons per family | 3 |
| Alpha | 0.05 |

| Tukey's multiple comparisons test | Mean Diff. | 95.00% CI of diff. | Significant? | Summary | Adjusted P Value |  |
| --- | --- | --- | --- | --- | --- | --- |
| 4 vs. 8 | 1.333 | -6.117 to 8.783 | No | ns | 0.8507 | A-B |
| 4 vs. 15 | -35.97 | -43.42 to -28.52 | Yes | **** | <0.0001 | A-C |
| 8 vs. 15 | -37.3 | -44.75 to -29.85 | Yes | **** | <0.0001 | B-C |

| Test details | Mean 1 | Mean 2 | Mean Diff. | SE of diff. | n1 | n2 | q | DF |
| --- | --- | --- | --- | --- | --- | --- | --- | --- |
| 4 vs. 8 | 14.27 | 12.93 | 1.333 | 2.428 | 3 | 3 | 0.7766 | 6 |
| 4 vs. 15 | 14.27 | 50.23 | -35.97 | 2.428 | 3 | 3 | 20.95 | 6 |
| 8 vs. 15 | 12.93 | 50.23 | -37.3 | 2.428 | 3 | 3 | 21.73 | 6 |

**MUFA in TAG at -N**

| Data sets analyzed | A-C |
| --- | --- |
|  |  |
| ANOVA summary |  |
| F | 35.8 |
| P value | 0.0005 |
| P value summary | *** |
| Significant diff. among means (P < 0.05)? | Yes |
| R square | 0.9227 |
|  |  |
| Brown-Forsythe test |  |
| F (DFn, DFd) | 1.263 (2, 6) |
| P value | 0.3484 |
| P value summary | ns |
| Are SDs significantly different (P < 0.05)? | No |

| ANOVA table | SS | DF | MS | F (DFn, DFd) | P value |
| --- | --- | --- | --- | --- | --- |
| Treatment (between columns) | 12598 | 2 | 6299 | F (2, 6) = 35.80 | P=0.0005 |
| Residual (within columns) | 1056 | 6 | 175.9 |  |  |
| Total | 13654 | 8 |  |  |  |

| Number of families | 1 |
| --- | --- |
| Number of comparisons per family | 3 |
| Alpha | 0.05 |

| Tukey's multiple comparisons test | Mean Diff. | 95.00% CI of diff. | Significant? | Summary | Adjusted P Value |  |
| --- | --- | --- | --- | --- | --- | --- |
| 4 vs. 8 | 1.9 | -31.33 to 35.13 | No | ns | 0.9832 | A-B |
| 4 vs. 15 | -78.4 | -111.6 to -45.17 | Yes | *** | 0.0009 | A-C |
| 8 vs. 15 | -80.3 | -113.5 to -47.07 | Yes | *** | 0.0008 | B-C |

| Test details | Mean 1 | Mean 2 | Mean Diff. | SE of diff. | n1 | n2 | q | DF |
| --- | --- | --- | --- | --- | --- | --- | --- | --- |
| 4 vs. 8 | 77.33 | 75.43 | 1.9 | 10.83 | 3 | 3 | 0.2481 | 6 |
| 4 vs. 15 | 77.33 | 155.7 | -78.4 | 10.83 | 3 | 3 | 10.24 | 6 |
| 8 vs. 15 | 75.43 | 155.7 | -80.3 | 10.83 | 3 | 3 | 10.49 | 6 |

**PUFA in polar fraction at -N**

| Data sets analyzed | A-C |
| --- | --- |
|  |  |
| ANOVA summary |  |
| F | 74.96 |
| P value | <0.0001 |
| P value summary | **** |
| Significant diff. among means (P < 0.05)? | Yes |
| R square | 0.9615 |
|  |  |
| Brown-Forsythe test |  |
| F (DFn, DFd) | 0.5360 (2, 6) |
| P value | 0.6107 |
| P value summary | ns |
| Are SDs significantly different (P < 0.05)? | No |

| ANOVA table | SS | DF | MS | F (DFn, DFd) | P value |
| --- | --- | --- | --- | --- | --- |
| Treatment (between columns) | 1636 | 2 | 817.8 | F (2, 6) = 74.96 | P<0.0001 |
| Residual (within columns) | 65.46 | 6 | 10.91 |  |  |
| Total | 1701 | 8 |  |  |  |

| Number of families | 1 |
| --- | --- |
| Number of comparisons per family | 3 |
| Alpha | 0.05 |

| Tukey's multiple comparisons test | Mean Diff. | 95.00% CI of diff. | Significant? | Summary | Adjusted P Value |  |
| --- | --- | --- | --- | --- | --- | --- |
| 4 vs. 8 | -1.9 | -10.17 to 6.375 | No | ns | 0.7699 | A-B |
| 4 vs. 15 | 27.6 | 19.33 to 35.87 | Yes | *** | 0.0001 | A-C |
| 8 vs. 15 | 29.5 | 21.23 to 37.77 | Yes | **** | <0.0001 | B-C |

| Test details | Mean 1 | Mean 2 | Mean Diff. | SE of diff. | n1 | n2 | q | DF |
| --- | --- | --- | --- | --- | --- | --- | --- | --- |
| 4 vs. 8 | 43.17 | 45.07 | -1.9 | 2.697 | 3 | 3 | 0.9963 | 6 |
| 4 vs. 15 | 43.17 | 15.57 | 27.6 | 2.697 | 3 | 3 | 14.47 | 6 |
| 8 vs. 15 | 45.07 | 15.57 | 29.5 | 2.697 | 3 | 3 | 15.47 | 6 |

**PUFA in TAG fraction at -N**

| Data sets analyzed | A-C |
| --- | --- |
|  |  |
| ANOVA summary |  |
| F | 10.21 |
| P value | 0.0117 |
| P value summary | * |
| Significant diff. among means (P < 0.05)? | Yes |
| R square | 0.7729 |
|  |  |
| Brown-Forsythe test |  |
| F (DFn, DFd) | 0.9594 (2, 6) |
| P value | 0.435 |
| P value summary | ns |
| Are SDs significantly different (P < 0.05)? | No |

| ANOVA table | SS | DF | MS | F (DFn, DFd) | P value |
| --- | --- | --- | --- | --- | --- |
| Treatment (between columns) | 4697 | 2 | 2348 | F (2, 6) = 10.21 | P=0.0117 |
| Residual (within columns) | 1380 | 6 | 230 |  |  |
| Total | 6077 | 8 |  |  |  |

| Number of families | 1 |
| --- | --- |
| Number of comparisons per family | 3 |
| Alpha | 0.05 |

| Tukey's multiple comparisons test | Mean Diff. | 95.00% CI of diff. | Significant? | Summary | Adjusted P Value |  |
| --- | --- | --- | --- | --- | --- | --- |
| 4 vs. 8 | 3.167 | -34.83 to 41.16 | No | ns | 0.9648 | A-B |
| 4 vs. 15 | 49.97 | 11.97 to 87.96 | Yes | * | 0.0161 | A-C |
| 8 vs. 15 | 46.8 | 8.804 to 84.80 | Yes | * | 0.0215 | B-C |

| Test details | Mean 1 | Mean 2 | Mean Diff. | SE of diff. | n1 | n2 | q | DF |
| --- | --- | --- | --- | --- | --- | --- | --- | --- |
| 4 vs. 8 | 135.3 | 132.1 | 3.167 | 12.38 | 3 | 3 | 0.3616 | 6 |
| 4 vs. 15 | 135.3 | 85.33 | 49.97 | 12.38 | 3 | 3 | 5.706 | 6 |
| 8 vs. 15 | 132.1 | 85.33 | 46.8 | 12.38 | 3 | 3 | 5.345 | 6 |

**Total lipids in polar fraction at -N**

| Data sets analyzed | A-C |
| --- | --- |
|  |  |
| ANOVA summary |  |
| F | 0.1067 |
| P value | 0.9005 |
| P value summary | ns |
| Significant diff. among means (P < 0.05)? | No |
| R square | 0.03434 |
|  |  |
| Brown-Forsythe test |  |
| F (DFn, DFd) | 0.1277 (2, 6) |
| P value | 0.8824 |
| P value summary | ns |
| Are SDs significantly different (P < 0.05)? | No |

| ANOVA table | SS | DF | MS | F (DFn, DFd) | P value |
| --- | --- | --- | --- | --- | --- |
| Treatment (between columns) | 14.48 | 2 | 7.241 | F (2, 6) = 0.1067 | P=0.9005 |
| Residual (within columns) | 407.3 | 6 | 67.88 |  |  |
| Total | 421.8 | 8 |  |  |  |

| Number of families | 1 |
| --- | --- |
| Number of comparisons per family | 3 |
| Alpha | 0.05 |

| Tukey's multiple comparisons test | Mean Diff. | 95.00% CI of diff. | Significant? | Summary | Adjusted P Value |  |
| --- | --- | --- | --- | --- | --- | --- |
| 4 vs. 8 | 1.733 | -18.91 to 22.37 | No | ns | 0.9643 | A-B |
| 4 vs. 15 | -1.367 | -22.01 to 19.27 | No | ns | 0.9776 | A-C |
| 8 vs. 15 | -3.1 | -23.74 to 17.54 | No | ns | 0.8916 | B-C |

| Test details | Mean 1 | Mean 2 | Mean Diff. | SE of diff. | n1 | n2 | q | DF |
| --- | --- | --- | --- | --- | --- | --- | --- | --- |
| 4 vs. 8 | 75.8 | 74.07 | 1.733 | 6.727 | 3 | 3 | 0.3644 | 6 |
| 4 vs. 15 | 75.8 | 77.17 | -1.367 | 6.727 | 3 | 3 | 0.2873 | 6 |
| 8 vs. 15 | 74.07 | 77.17 | -3.1 | 6.727 | 3 | 3 | 0.6517 | 6 |

**Total lipids in TAG fraction at -N**

| Data sets analyzed | A-C |
| --- | --- |
|  |  |
| ANOVA summary |  |
| F | 7.106 |
| P value | 0.0262 |
| P value summary | * |
| Significant diff. among means (P < 0.05)? | Yes |
| R square | 0.7031 |
|  |  |
| Brown-Forsythe test |  |
| F (DFn, DFd) | 0.1174 (2, 6) |
| P value | 0.8912 |
| P value summary | ns |
| Are SDs significantly different (P < 0.05)? | No |

| ANOVA table | SS | DF | MS | F (DFn, DFd) | P value |
| --- | --- | --- | --- | --- | --- |
| Treatment (between columns) | 13036 | 2 | 6518 | F (2, 6) = 7.106 | P=0.0262 |
| Residual (within columns) | 5504 | 6 | 917.3 |  |  |
| Total | 18539 | 8 |  |  |  |

| Number of families | 1 |
| --- | --- |
| Number of comparisons per family | 3 |
| Alpha | 0.05 |

| Tukey's multiple comparisons test | Mean Diff. | 95.00% CI of diff. | Significant? | Summary | Adjusted P Value |  |
| --- | --- | --- | --- | --- | --- | --- |
| 4 vs. 8 | 4.233 | -71.64 to 80.11 | No | ns | 0.984 | A-B |
| 4 vs. 15 | -78.53 | -154.4 to -2.659 | Yes | * | 0.0438 | A-C |
| 8 vs. 15 | -82.77 | -158.6 to -6.893 | Yes | * | 0.0356 | B-C |

| Test details | Mean 1 | Mean 2 | Mean Diff. | SE of diff. | n1 | n2 | q | DF |
| --- | --- | --- | --- | --- | --- | --- | --- | --- |
| 4 vs. 8 | 240.3 | 236.1 | 4.233 | 24.73 | 3 | 3 | 0.2421 | 6 |
| 4 vs. 15 | 240.3 | 318.8 | -78.53 | 24.73 | 3 | 3 | 4.491 | 6 |
| 8 vs. 15 | 236.1 | 318.8 | -82.77 | 24.73 | 3 | 3 | 4.733 | 6 |
