# Supplementary material for: Temperature-Dependent Lipid Accumulation in the Polar Marine Microalga Chlamydomonas malina RCC2488
Source: Front Plant Sci. 2020 Dec 23;11:619064. doi: 10.3389/fpls.2020.619064 (PMC7785989; doi:10.3389/fpls.2020.619064)
Supplement: Supplementary file 2 [file Table_2.docx]

Temperature-dependent lipid accumulation in the polar marine microalga *Chlamydomonas malina* RCC2488

Daniela Morales-Sánchez ^1,2*^, Peter S. C. Schulze ^2,3^, Viswanath Kiron^2^, Rene H. Wijffels ^2,4^

^^

***Correspondence:**Daniela Morales-Sánchez

[daniela.morales-sanchez@uit.no](mailto:daniela.morales-sanchez@uit.no)


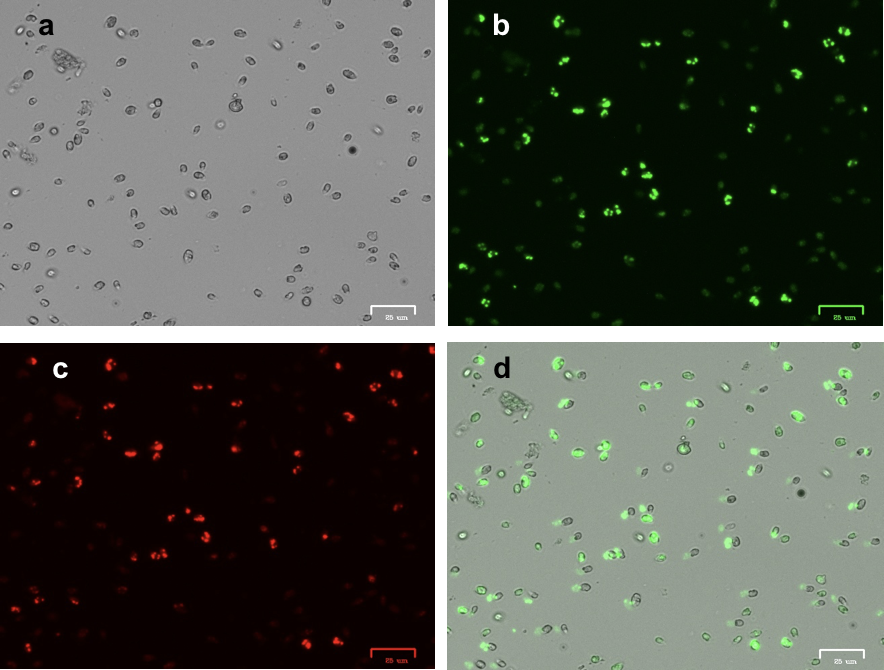


**Figure S1**. Nonpolar lipid accumulation in *C. malina* at 4 ºC subject to nitrogen deprivation. Bright field (a), green filter (b), red filter (c), and green filter merged with bright field (d). The images shown are representative of typical cells in the samples. Nile red fluorescence was pseudo-colored red and green for visualization. Scale bars equal 25 µm.
